# A topological method of generating action potentials and electroencephalography oscillations in a surface network

**DOI:** 10.1098/rsos.241977

**Published:** 2025-05-28

**Authors:** Siddhartha Sen

**Affiliations:** ^1^ Trinity College Dublin, Dublin, Ireland

**Keywords:** electroencephalography, oscillations, network

## Abstract

The brain is a source of continuous electrical activity, which includes one-dimensional voltage pulses (action potentials) that propagate along nerve fibres, transient localized oscillations and persistent surface oscillations in five distinct frequency bands. However, a unified theoretical framework for modelling these excitations is lacking. In this article, we provide such a framework by constructing a special surface network in which all observed brain-like signals, including surface oscillations, can be generated by topological means. Analytic expressions for all these excitations are found, and the values of the five frequency bands of surface oscillations are correctly predicted. It is shown how input signals of the system produce their own communication code to encode the information they carry and how the response output propagating signals produced carry this input information with them and can transfer it to the pathways they traverse as a non-transient topological memory structure of aligned spin-half protons. It is conjectured that the memory structure is located in the insulating sheaths of nerve fibres and is stable only if the pathways between the assembly of neurons, which represents a memory structure, include loops. The creation time and size of memory structures are estimated, and a memory-specific excitation frequency for a memory structure is identified and determined, which can be used to recall memories.

## 1. Introduction

In this article, we introduce a special topological surface network with surface electrons and protons that captures a key global feature of the brain. An analysis of the network shows that it can generate all brain-like signals and store memories as aligned proton spin structures located in the insulating myelin sheaths of nerve fibres.

The brain consists of a vast number ($N\approx 10^{11}$) of neurons [1]. Each neuron has multiple signal-receiving stations, protrusions, called dendrites. Signals entering the dendrites are processed as they enter the neuron, and if they cross a certain threshold energy, a processed signal or action potential is generated that exits the neuron, as a one-dimensional voltage pulse via its one single signal outlet nerve fibre, the axon. The axon terminal of one neuron passes on the signal it carries to the dendrite of another neuron in the form of a cloud of specialized molecules called neurotransmitters. There is a gap of about $10^{-6}\;cm$ between the axon of one neuron and the receiving dendrite terminal of another neuron, called a synapse. The membranes that cover the neurons and axons contain ions, and a voltage difference between the inner and outer surfaces of the membranes is observed. Protein gates on the axon membrane connect its inner and outer surface and open and close under appropriate conditions to allow ions to flow between them. It is believed that electrical signals generated by the brain are due to ion flows using properties of these ion gates [1].

A variety of electrical activities are observed in the brain. They include different types of one-dimensional voltage pulses that propagate along the axons, localized transient oscillations, as well as the ever-present brain surface voltage oscillations revealed by electroencephalography (EEG) measurements [2] on the scalp. The EEG oscillations are observed to belong to five frequency bands, called the delta (0.5−3 Hz), theta (3−8 Hz), alpha (8−12 Hz), beta (12−30 Hz) and gamma (30−42+ Hz) waveforms. It is established that action potentials play an essential role in the functioning of the brain. They initiate motor or emotional responses, while EEG waveforms seem to respond whenever action potentials are generated, but the precise link between them is not clear.

Understanding the autonomous functioning of this vast and complex system is a formidable task. But remarkable progress in unravelling key features of the way the brain functions has been made. Observational neuroscience is progressing at a rapid pace using a variety of innovative experimental techniques to suggest how memories are created, how they may be manipulated, how they may be stored in collections of special engram cells [3–5] and how memories may be space and time tagged [5,6]. Theoretical neuroscience too is progressing [7] driven by new ideas of theoretical representations of the brain [8] and increased computational power. Methods to theoretically model behaviour, discover new excitations and suggested ways to understand complex brain events and store memory [9] are emerging [10].

However, there are major conceptual and theoretical problems that remain unaddressed. Areas of concern include the lack of theories that allow signals to carry information [1], the lack of a theoretical understanding of how memories are stored [11], and the lack of understanding of how the properties of observed EEG waveforms are generated [2].

Observations strongly suggest that sensory input signals to the brain carry with them information that is processed to give us our sensory experiences. If the brain regards some incoming information as important, then it is stored as long-term memory. The process of memory creation has been very well studied [12] but where they are stored is still under investigation. A current suggestion is that memories may be stored in the pathways between special engram cells [3].

Interactions between incoming signals and memory are essential for our brain to function. Such interactions allow us to identify people we know, to avoid dangerous places, help us to remember and return to where we live and to engage in conversations. Yet theoretical neuroscience cannot address this issue. In current theoretical neuroscience, memory information is fed into the system by special input signals and retrieved by special input cues. They are not directly related to all incoming input signals. Brain functions are currently modelled by introducing interaction between individuals and populations of neurons that are either always excitatory or inhibitory in linear networks that are chosen to reflect the observed connectivity of the brain or that describe important pathways between brain organs. Simulation of brain activities is carried out by introducing ‘integrate and fire’ input signals [7] that do not and cannot carry biological information. Thus, two important features of the brain are ignored. Signals do not carry information, so no theoretical link between signals and memory is possible, and the constant feedback interactions and information exchange between signals and memories that occurs in the brain are missing.

Another major theoretical gap is the absence of understanding the origin of EEG waveforms and the way they interact with other brain signals. A current suggestion [13] that EEG waveforms are created by dipole current loops on dendrites cannot explain the observed properties of EEG waveforms. This gap means that there is no theoretical way to describe the interaction of EEG waveforms with memory that is observationally seen to occur [14].

We will prove that current theoretical signals cannot carry information. This means that even if the integrate and fire signals are replaced by realistic signals, such as the membrane ion flow model of Hodgkin–Huxley [1] signals or the acoustic membrane signals of Jackson and Heimburg [15], they still would not provide the information loop between signals and memory required to understand cognitive functions of the brain, as even these signals cannot carry information.

Let us prove this result for the Hodgkin–Huxley [1] ion membrane model and the membrane acoustic soliton model [15]. The ion membrane model, as pointed out by Scott [1], is a dissipative wave model. In this, inflows and outflows of ions between the inner and outer membrane surfaces of an axon lead to the injection of energy in the centre of the axon tube in the form of a voltage gradient. The voltage gradient produced is an energy source and produces a dissipative ohmic current. The process of ion flow is triggered when an incoming signal crosses a voltage threshold that leads to the opening of membrane gates. No other condition is necessary. Thus, the signals produced propagate by following a causal cycle of energy injection and dissipation. Such a dissipative wave cannot carry information regarding its creation as causal cycles of energy injection and dissipation that depend only on membrane properties and not on the initial cause of the signal. In modelling the process of energy injection, the membrane’s inner and outer surface ion distributions are found by using thermodynamics. However, thermodynamic results are independent of the history of how the system parameter values are reached. The conclusion is that signals in dissipative wave theories follow a causal cycle that does not allow signals produced to carry information about their creation. The observed link between signal information and memory is missing in such theories. Scott, who was a distinguished applied mathematician, compares nerve signal propagation with the dissipative mechanism of a burning candle as discussed by the great scientist Michael Faraday in his Christmas lectures [1]. The analogy is that a burning candle is also a dissipative process in which melted wax reaches the tip of the wick by capillary action, becomes a vapour and injects energy that is dissipated by the burning flame. This causal cyclic dissipative process of the burning candle carries no information regarding how the process was started. It is a coherent process that continues as long as the energy source and the dissipative processes are operative. It has no memory of how the process was started.

The acoustic soliton approach of Jackson [15] unfortunately faces the same problem. Acoustic solitons discovered are non-dissipative, non-topological excitations that are produced as membrane acoustic waves. They depend on the axon membrane’s elastic properties and the fact that the fluid inside the axon undergoes a phase transition when compressed. Thus, a key step for producing soliton signals involves thermodynamic properties and is thus independent of their history, so the soliton waves produced cannot carry information of their creation.

These results impact the capabilities of theoretical approaches to understand memory formation and place limitations on current approaches to model cognitive brain functions [16–18].

Thus, a radical deviation from existing approaches is needed. Our aim is to show that there is such an approach. This is demonstrated by constructing a theoretical surface network which creates its own communication code, produces the range of brain excitations observed, including EEG waveforms, where signals generated clearly carry information regarding their creation, and a mechanism, based on the laws of physics, is suggested for the signals to transfer the information they carry to form stable memories. In the network, memories are retrieved by a resonance excitation method, as it is shown that the memory structure created has a memory-specific excitation frequency. Signals produced can interact with each other, and they have an information exchange loop with memory. However, the method of signal generation suggested is unconventional.

### 1.1. Topological ideas and Riemann surfaces

The stated properties of the special surface network come from its topological features. Topology is a mathematical discipline in which objects that can be deformed into each other by smooth deformations are regarded as the same. Thus, a soccer ball and a rugby ball are topologically identical.^1^

The surface network we chose has topological properties. It exactly captures the topological connectivity of the axons of the brain as a surface. We will prove this result. It can produce all brain-like signals in response to local topology-changing deformations of a subunit, only if the surface is described by a specific algebraic equation that uniquely defines a special Riemann surface. Riemann surfaces provide a geometric description of any polynomial equation in two complex variables [20]. Thus, a special polynomial equation is represented by a special Riemann surface. We will explain why a special Riemann surface is required for signal generation. The final property of the surface is that it must have charged surface particles of spin-half.

The presence of surface spin-half particles introduces an additional essential layer of topology to the surface. Without surface spin-half particles, none of our results would hold. The topology due to spin-half particles comes from the ability of such particles to arrange themselves in topologically distinct patterns because spin-half particles have magnetic properties.

The topological structure due to spin is called a spin structure [21]. The topological connectivity of the surface network can be described by an integer g called the genus of the Riemann surface, while its spin topological structure can be described by another integer W called its topological spin number. These numbers will be defined shortly. Signals are generated by local topology-changing surface deformations of subunits of the Riemann surface that deform the topology numbers (k,w), which describe the subunits’ connectivity and spin structure, to the values (k=0,w=0) which describe a spherical surface. Such a process generates k non-dissipative one-dimensional voltage pulses, called solitons.

### 1.2. A surface connectome is a Riemann surface

**Figure 1.**
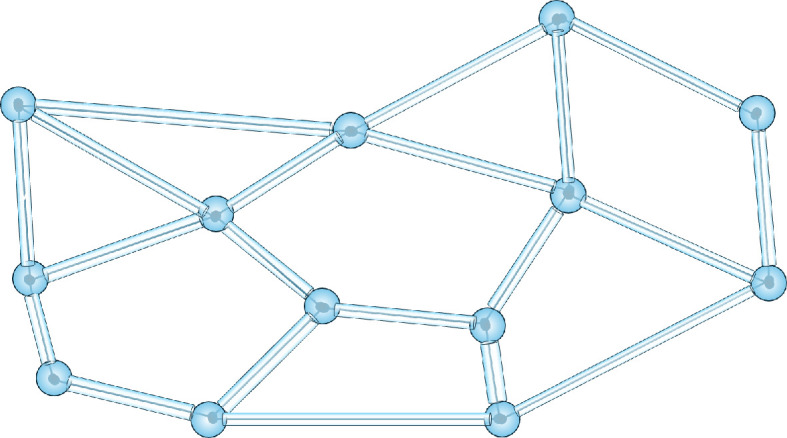
A part of a brain connectome represented by a surface, where the lines of the connectome are replaced by tubes and junction points (neurons) are replaced by junction spheres. See text for details.

We now prove that an exact representation of the topological connectivity features of the brain’s axons by a mathematical Riemann surface is possible. Consider a hypothetical brain connectome^2^ that describes the axon connectivity features of a brain as a one-dimensional network, embedded in three dimensions, where each neuron is a nodal point of the network and the lines are axons. The network is enormously complicated and unknown to the human brain. We now replace each line of the connectome with a tube and each nodal point with a suitable nodal surface (figure 1).

**Figure 2.**
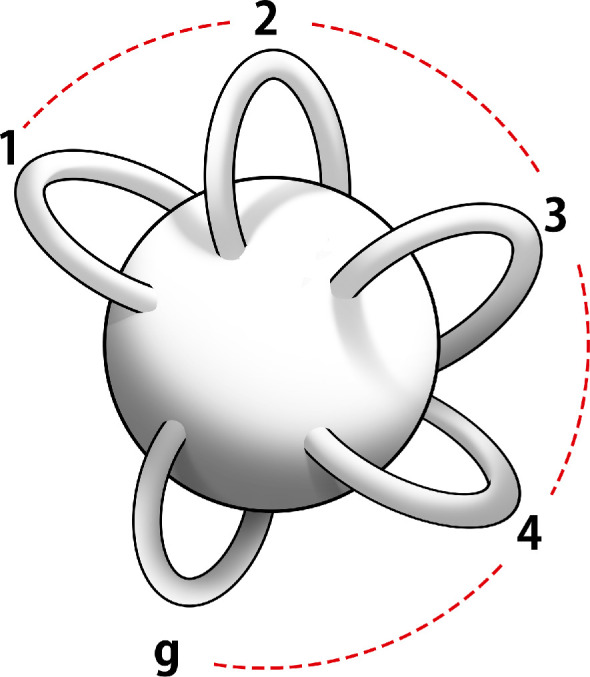
Genus g surface for *g* = 5.

**Figure 3.**
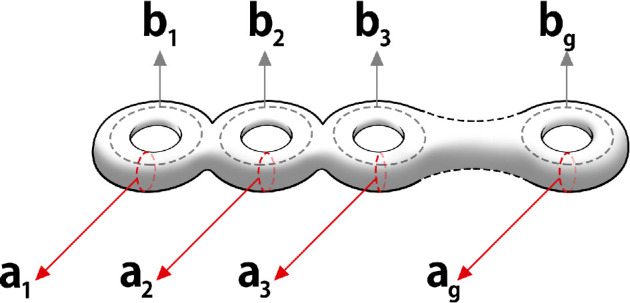
Marked Riemann surface with loop coordinates.

This step seems to make the original network even more complex, but a remarkable result from the mathematical discipline of topology [22] now comes to our aid. The topological result tells us that any closed surface, no matter how intricate or complex, is topologically equivalent to a sphere with g handles attached to it (figure 2) or an orderly collection of g doughnuts [23] (figure 3).

The only topological difference between any two surfaces is in the value of one integer number g called its genus. This number, the genus g, completely captures the topological connectivity of a surface. If we assume, as we do, that the surface is smooth, then it can be represented by a mathematical surface known as a Riemann surface of genus g.

Thus, from the classification theorem, it follows that the topological connectivity of any brain connectome can be exactly represented as a smooth Riemann surface of unknown genus g, and thus is a geometric representation of an algebraic equation.

### 1.3. Signal generation and the Riemann theta function

We next explain how brain signals can be generated by topological means and then sketch why such signals are solutions of the nonlinear Schrödinger equation. A topological way to produce soliton pulses was discovered by Mumford [24], who showed that soliton solutions of the nonlinear Korteweg–De Vries (KdV) differential equation could be generated by local topology-changing deformation of a Riemann surface with spin structure.

We need to modify Mumford’s ideas in two ways so that the approach can produce the range of brain-like activity we are interested in and at the same time co-produce EEG-like waveforms. These requirements can be met by an extension of Mumford’s idea, encapsulated as the dynamical law of the network.

The second modification introduced is a restriction. Thus, unlike Mumford, the network surface cannot be an arbitrary Riemann surface but must be one that represents a specific algebraic equation known as the hyperelliptic equation, which is defined by the equation $w^{2}=\Pi_{i=1}^{\left(2g+2\right)}(z-x_{i})$ where the roots of the polynomial are either real or appear as complex conjugate pairs. Recall if z=a+ib, the $\bar{z}=a-ib$ are a complex conjugate pair. The surface must also have charged spin-half particles, such as electrons and protons. This restriction allows the system to produce the wide variety of brain-like signals we want. The mathematical reason for this restriction is explained in Arberello [25].

The surface network can be visualized as a membrane cover of the individual neurons of the brain with the topological connectivity of the brain’s connectome. Thus, the Riemann surface tubes are axons, its nodal junction regions are the location of neurons, and the points where the tubes enter a junction are the location of dendrites. We will use these descriptive terms.

### 1.4. Input pinch deformations and electroencephalography waveforms

The first step is to explain how the topology of a subunit is changed by local input signals, called pinch deformations. Pinch deformations reduce the circumference of an axon tube at a point to zero and thus change the topology of the system. Consider a subunit of genus k<g and spin structure w<W. Pinch deformations of this subunit reduce its topology numbers from (k,w) to (0,0). The topology numbers (k=0,w=0) as stated before, describe a topological sphere. When this happens, we will see k one-dimensional soliton voltage pulses that exit through the axons that connect the subunit to other subunits of the surface, and each soliton pulse carries with it a topological spin number phase $e^{i\pi w}$. The excitations carry away the topology numbers of the subunit as well as the pinch deformation that produced them. Thus, signals carry information encoded in the system’s own communication code given by pinch deformation parameter values. We have yet to define the spin-topology number w. This will be done shortly.

The process described implies that whenever signals are produced by pinch deformations, topological spheres are created. It is expected that these topological sphere surfaces will have voltage oscillations. We identify these spheres as individual neuron surfaces^3^ and the surface oscillations of an assembly of neurons as the source of the observed scalp EEG waveform voltage oscillations.

### 1.5. Signals are solutions of a nonlinear equation

We next explain the surprising result that all signals generated by the pinch deformation of subunits must be solutions of a special nonlinear differential equation called the nonlinear Schrödinger equation.

To do this, we first introduce an appropriate function to describe the network’s response to pinch deformations. Recall that if we are interested in the oscillations of a circle, we introduce sine and cosine functions that have as variables the angle of the circle. For a Riemann surface, there is an analogous function called the Riemann theta function. The variables that define it depend on, as expected, the variables of the Riemann surface. Unlike the sine and cosine functions that depend on the one variable that defines a circle, the number of variables in the Riemann theta function is more than what is necessary. This means the variables cannot be freely chosen. The Riemann theta function has to satisfy constraints. A mathematical result tells us that the constraint is a nonlinear identity.

We now have the concepts required to state the dynamical law for the network. It requires that both input pinch deformations and the responses they produced, described in terms of Riemann theta functions, must be compatible with the Riemann surface structure even in the pinch limit. It is then shown that the nonlinear constraints, in the limit of a pinch deformation, become a set of nonlinear Schrödinger equations, only if it represents a special algebraic equation. Then the response to pinch deformations is Riemann theta functions that satisfy the nonlinear Schrödinger differential equation. They exist, and their analytic forms are known. The technical details are omitted [24].

We now postulate the dynamical law of the network. The dynamical law has two parts. The first part notes that all deformations of the Riemann surface are automatically transferred to the deformation of the variables of the Riemann theta function associated with it, as these are related, as explained already.

The second part makes use of a mathematical theorem that states that a Riemann theta function represents a Riemann surface only if it satisfies a nonlinear identity called the Fay trisecant identity [24,27]. We will explain the intuitive basis of this requirement. A more technical argument will be sketched later.

The dynamical law thus requires that when the Riemann surface is deformed, the Riemann theta function with its variables automatically deformed must continue to satisfy the Fay identity. Since, in the pinch deformation limit, the Fay identities [28] become a set of nonlinear Schrödinger equations [28], the Riemann theta responses to pinch deformations must be solutions of the nonlinear Schrödinger equation. But the nonlinear Schrödinger result holds only when the Riemann surface represents a hyperelliptic equation.

Thus, the dynamical law tells us that input pinch deformations produce outputs that are solutions of the nonlinear Schrödinger equation. Solutions of the nonlinear Schrödinger equation can represent a wide variety, possibly all, observed brain-like signals, and their analytic forms are known, and the solutions carry pinch deformation information. Thus, the claim is that any given observed brain-like signals, no matter how they are produced, can be fitted by choosing suitable pinch deformation parameters. We will give an example of such a fit in a later section.

### 1.6. How signals produce memory

We sketched why voltage soliton pulse signals produced by pinch deformations are described in terms of the Riemann theta function, where the Riemann theta function variables contain the pinch deformation information, and the resulting signals are solutions of the nonlinear Schrödinger equation.

We now show how these transient soliton signal voltage pulse signals can transfer the information they carry to form non-transient memory structures of aligned surface spin-half particles, in the pathways they traverse. This information transference process happens because of the laws of electromagnetism. Moving pulses carry charge and thus generate a helical transient magnetic field [29],^4^ which acts on the magnetic spin-half surface particles, aligning them to form a non-transient structure that captures the magnetic field profile and hence, it contains all deformation details responsible for creating the voltage pulse signal.

This helical spin structure, if stable, is a substrate for memory.^5^ We show the structure has a signal-specific excitation frequency. This means that long-term memories can be recalled by a resonance excitation method by a suitable excitation frequency. We will, in a later section, discuss the requirements to create stable memories and derive a formula for their creation time and size.

We have given an intuitive account of how the surface network can produce signals and store memory. Our next step is to add in the missing mathematical details.

## 2. Outline of the paper

First, we give an outline of the paper. After that, we address mathematical issues. In the next section, the mathematical variables that define a Riemann surface and its associated Riemann theta functions are introduced, and the spin topology number W is defined. We then explain why the Riemann theta function has to satisfy the Fay trisecant identity at a more technical level. This is followed by postulating a dynamical law of the network in formal terms. We then define the input signals of the network as local pinch deformations.^6^ The analytic form of moving voltage soliton pulse signals is known. This means we can determine the helical magnetic fields they generate and then, from standard methods of physics, determine the spin-aligned memory structure of spin-half surface particles in response to the transient magnetic field of the moving solitons. We are able to theoretically show that each memory structure will have its own characteristic excitation frequency and determine the frequency values they have. We then estimate the time required to create memory structures. The creation time estimate comes from the conditions needed for the memory structure to be stable. We then discuss how memories can be retrieved by exploiting their excitation frequencies.

After that, we then turn to discuss EEG waveform generation. We show that they can be identified as sphere surface voltage oscillations that are produced whenever pinch deformation-generated signals are generated by pinch deformations. We theoretically predict that these oscillations have five oscillation frequency bands with amplitude values inversely related to their frequencies.

Following these general results, we shift our attention to a special subclass of tiling solutions of the wave equation known as dihedral tiling. These solutions are special as they form a complete set of solutions. There are an infinite number of them, and linear combinations of these solutions can be used to represent all other waveforms as well as arbitrary functions on the surface of the sphere. For the rest of the paper, we restrict ourselves to these solutions and use their special mathematical features to develop the mathematical tools required to analyse the interaction of EEG waveforms with other brain excitations and with memory. With these theoretical results in place, we proceed to model and explain a sequence of brain excitations that are observed during deep sleep.

We end by listing the testable predictions of the approach, drawing attention to some of its special features that might provide a different way to understand brain functions, and outlining future work. Our most significant prediction is that memories are encoded in aligned helical surface spin-half protons located in the insulating myelin sheaths of axons. We provide evidence that supports the conjecture. We also predict that a topologically stable memory structure is an assembly of neurons connected together by axon pathways that have a non-zero spin topology number. This means the axon pathways must have loops. The predicted memory structure is similar to an engram.

Let us define the variables we need.

## 3. Riemann surfaces

### 3.1. Riemann surface and theta function variables

A Riemann surface is a smooth topological surface. It is defined by topological coordinates that describe its connectivity and a mathematical object, called one form, that describes its smoothness. We define these coordinates and the smoothness measure. We then show how these Riemann surface variables are used to define the variables of its associated Riemann theta function.

As a Riemann surface represents an algebraic equation, it is expected that its topological coordinates and its smoothness measure must be constructible from its associated algebraic equation, in our case the hyperelliptic equation. We will briefly indicate how this can be done. This sketch makes the conceptual features of the approach clear.

The topological connectivity of the Riemann surface is captured by 2g loops, as shown in figure 3. They are g loops $a_{i}$ that go around the tubes of the Riemann surface as shown, while the g loops $b_{i}$ circle round loops of the doughnuts, as shown. The smoothness properties of a Riemann surface are captured by a set of objects known as a one-form. A one-form can be written locally as f(z)dz, where z is a complex number that represents a surface point. It is an object that can be integrated along a line or loop. Riemann proved that a genus g Riemann surface has exactly g smooth one-forms. We write them as $\omega (z{)}_{j}dz$ where the functions $\omega (z{)}_{j}$ are smooth functions, where z is a complex variable that represents a point on the genus g Riemann surface. These one forms can be integrated over the loops $\left(a_{i},b_{i}\right)$ of figure 3, to give complex numbers. Riemann normalized these one-forms so that $\int_{a_{i}}\omega (z{)}_{j}dz=\delta_{ij}$ where $\delta_{ij}=1$, when i=j but is zero otherwise. Riemann then proved that $\int_{b_{i}}\omega (z{)}_{j}dz=\Omega_{ij}$ was a complex symmetric matrix and that its imaginary part entries are all positive. It is called the period matrix of the Riemann surface. This ends our mathematical description of a Riemann surface.

In our discussions, we consider a special Riemann surface of genus g that represents the hyperelliptic equation $w^{2}=P(z)=\Pi_{i=1}^{\left(2g+2\right)}(z-x_{i})$. We now briefly sketch how to construct the coordinates and one-forms of this genus g Riemann surface starting from the equation. The g one forms are defined by $\omega_{j}(z)dz=\frac{z^{j-1}dz}{\sqrt{P(z)}}$ and the 2g loops can be constructed from the location of the (2g+2) zeros of P(z). The details of the construction are in Mumford [24].

We next define the algebraic Riemann theta function. Its variables are constructed from the loops and one forms of its associated Riemann surface, and it also includes the spin structure of its Riemann surface. The spin structure variables in the Riemann theta function are defined by a set of 2g discrete parameters called the characteristics of the Riemann theta function. These parameters $\left(\alpha_{i},\beta_{i}\right)$ are associated with the $\left(a_{i},b_{i}\right)$ loops of the Riemann surface, and each one of them is either zero or half. It was proved by Mumford [24] that they capture the spin structure of its associated Riemann surface. We now define the Riemann theta function with characteristics that is associated with a genus g Riemann surface, $\Theta_{\alpha_{i},\beta_{i}}(\Omega_{ij},z_{i}),i=1,2,\ldots ,g$ by the expression


$$\Theta_{\left(\alpha_{i},\beta_{i}\right)}(\Omega_{ij},z_{i})=\sum_{n_{i}}e^{\left[i\pi \sum_{i,j}(n_{i}+\alpha_{i})\Omega_{ij}(n_{j}+\alpha_{j})+2i\pi (z_{i}+\beta_{i})(n_{i}+\alpha_{i})\right]}.$$

The variables $\left(z_{i},\Omega_{ij}\right)$ are constructed from the Riemann surface variables as explained, while the variables $\left(\alpha_{i},\beta_{i}\right)$ capture, as stated, the spin structure of the Riemann surface. They are discrete variables called characteristics. Each of them can be either zero or half, and each integer $n_{i}$ variable ranges between (±∞). The characteristics obey the following algebraic rules: $n\alpha_{i}=n\beta =0$ if n is an even integer and $n\alpha_{i}=\alpha_{i},n\beta_{i}=\beta_{i}$ when n is an odd integer, where multiplication by n means going round the $a_{i}$ loop n times for $\alpha_{i}$ and for going round the $b_{i}$ loop n times for $\beta_{i}$.

The additional set of g complex numbers $z_{i}$ are defined by $z_{j}=\int_{0}^{z}\omega (z)dz,i=1,2,\ldots ,g$ where z is an arbitrary point of the Riemann surface. Thus, a point on the Riemann surface produces g points in its Riemann theta function. We can now define our spin topology number $W=4\sum_{i=1}^{g}\alpha_{i}.\beta_{i}$. It is an integer and will play an important role in fixing the nature of EEG waveforms to be tiling oscillations.

**Figure 4.**
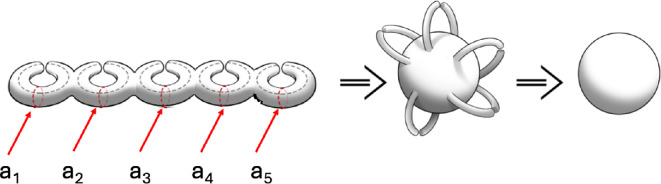
Degenerate Riemann surface due to pinching. The first figure has j doughnuts with j=5 tubes pinched, the second shows the same pinching now represented as five handles pinched and the final figure shows that these pinched systems are topological spheres.

We return to define a pinch deformation. Geometrically, it is a local topology-changing deformation in which the $a_{i}$ loop circumference shrinks to zero radius at a point (figure 4). We capture this geometric picture in an algebraic way as follows. The integral of a one-form $\omega_{j}(z)dz$ on the loop $a_{i}$ can be evaluated as $\int_{z=0}^{z}\omega_{j}(z)dz$ as z→0. The evaluation can be carried out by expanding $\omega_{j}(z)dz$ near z≈0 as a Taylor expansion of $\omega_{j}(z)dz$. The expansion coefficients define the distortion parameters. They will depend on the mathematical properties of the Riemann surface. A careful discussion of how this is done is explained in a separate work [19].

We next explain why the Riemann theta function has to satisfy a constraint for it to represent a Riemann surface.

### 3.2. The Fay identity and the dynamical law

It is known that the nature of a given Riemann surface of genus g is fixed by (6g−6) real parameters called its moduli [24]. These parameters capture the shape of the Riemann surface. However, the period matrix of a Riemann theta function has g(g+1) real parameters [24]. For g>2, the Riemann theta period matrix thus has more parameters than is required. This means that an arbitrary period matrix need not represent a Riemann surface. The parameters of the period matrix are not independent but have to satisfy constraints. But a mathematical result proves that if the Riemann theta function satisfies a constraint called the Fay trisecant identity [24], then the theta function will represent a Riemann surface. The Fay identity only holds if the Riemann surface has surface spin-half particles [27]. Hence, the presence of particles with spin half, on our surface network, turns the constraint problem into a well-defined requirement. We can now state the dynamical law as follows.

*Both input distortion signals and the surface responses they generate must preserve the Riemann structure of the surface*.

Thus, the dynamical law requires the Riemann theta function to satisfy the Fay trisecant identity, as this ensures that it continues to represent its associated Riemann surface even during deformation. In the pinch limit, the nonlinear Fay constraint becomes a set of nonlinear Schrödinger equations [28], provided the Riemann surface represents a hyperelliptic equation. For Riemann surfaces defined by other algebraic equations, other nonlinear differential equations emerge. Hence, we have the surprising result that for our Riemann surface, local pinch deformation generates excitations that are solutions of the nonlinear Schrödinger differential equation. This result follows from the dynamical law. The technical details are not important for our discussions and are omitted. They are given in Kalla [28]. Later, we will use the dynamical law to calculate properties of EEG waveforms. There, the requirement of preserving the underlying Riemann surface structure is enforced by insisting that only results that are conformal invariant, which means that have local scale invariance, are acceptable, since these transformations preserve the Riemann surface structure [20].

We have stated that soliton solutions and other excitations, created by pinch deformations, can produce brain-like signals. We now provide some observational evidence in support of this claim. Two sets of spike trains of action potential pulses were produced from a thalamic neuron of mice, one with two and the other with five spikes. These results were then fitted to the *N*-soliton solution of the nonlinear Schrödinger equation found by Previato [32]. The fits first select the number N of the solution, and then for the chosen N solution, the deformation parameter values, present in the solutions, are adjusted to fit the observational data. We reproduce these fits from our paper [19], where the data and fit details are given. The fits are very good. This result supports the claim that action potentials can be represented by solitons. Previato’s solution is written down in the electronic supplementary material.

**Figure 5.**
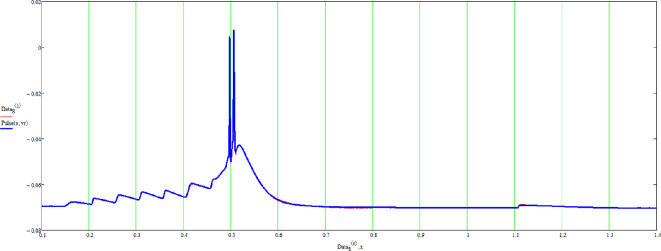
A firing thalamic neuron triggered by presynaptic simulation (blue) and the fit (red). See text for details.

**Figure 6.**
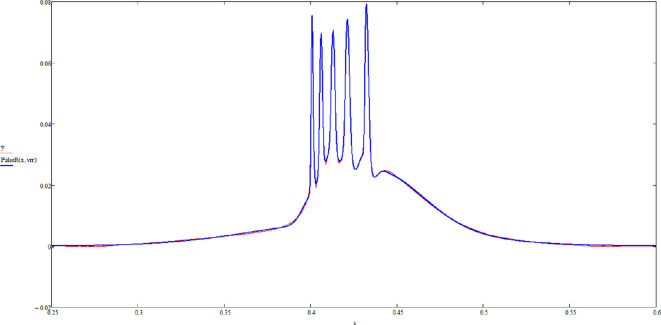
A firing thalamic neuron triggered by the injection of square voltage pulses (blue) and the fit (red). See text for details.

Let us present the examples. The same thalamic neuron in a mouse, as stated, is excited in two different ways. Once by presynaptic simulation, and then by the injection of square voltage spikes. The data curves are in blue, and the fits are in red (figures 5 and 6).

The example shows that multi-soliton solutions can indeed fit observed data and that signals produced in different ways can still be fitted by suitable pinch deformation. Finally, each pulse of the spike train is found to be different, as theoretically predicted.

Let us next turn to discuss the features of soliton pulse trains and describe them in a simple way. Consider a train of g soliton voltage pulses moving along the z axis. The jth soliton voltage pulse $V_{j}$ has a structure of the form $V_{j}(z,t)=e^{-i\pi W}V_{j}(a_{j}z-b_{j}t+c_{j}),j=1,2,\ldots ,g$, where $\left(a_{j},b_{j},c_{j}\right)$ are signal-specific pinch deformation parameters and W is the spin structure topology number. We will use this structure in our discussions. We should note that all variables are dimensionless. Thus, the position z is $\frac{z}{z_{0}}$, where $z_{0}$ is a length scale. Similarly, the time variable t is $\frac{t}{t_{0}}$, where $t_{0}$ is a time scale. Exact analytic results are given in Kalla [28] and Previato [32]. The deformation information carried by transient signals is transferred to create stable long-term memories in the pathways traversed by the signals. Let us explain how.

## 4. Memory

### 4.1. Signals align spins to form memory

Information transfer from pulse voltage signals to the pathways they traverse, which have surface spin-half particles on them, follows from the physics laws of electromagnetic theory [29]. Moving soliton pulses carry charge, and electromagnetic theory laws tell us that they produce transient helical magnetic fields around the direction of movement. These transient magnetic fields act on the surface spin-half particle, since they are magnetic, and align them to form a non-transient helical aligned spin structure which encodes the transient magnetic field details in it and is thus a memory. If the structure created is stable, it stores the information carried by the magnetic field of the moving soliton pulses as a memory.

**Figure 7.**
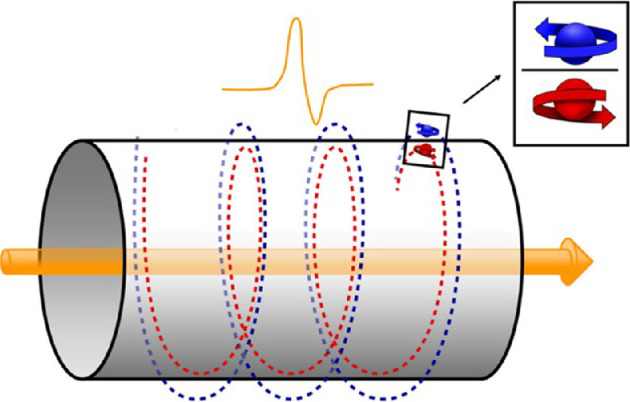
Theoretical helical field generated by a charged voltage pulse.

The transient magnetic field of a moving soliton voltage pulse has been shown to contain the same information as the voltage pulse that generates it by measuring the helical magnetic field (figure 7) produced by an action potential and then reconstructing the action potential [30].

Thus, the helical non-transient spin structure encodes the details of the transient magnetic field and thus the nature of the action potential that generated it. It is a memory. For memory to be useful, it should be stable over time, and a way of accessing the memory should be in place. We will show that this is possible, but for stable memory formation, certain conditions have to be met by the signals. We will discuss these stability conditions.

We now determine the memory structure, discuss its stability and then estimate its size and the time required to create a stable memory. Our first step is to replace the soliton voltage potentials with an equivalent charge distribution ρ, by using Poisson’s equation [29], and then use standard results of electromagnetic theory to determine the magnetic field generated by these moving charges [29].

Poisson’s equation relates voltage to charges by the equation $\nabla^{2}V_{j}(z,t)=4\pi \rho_{j}(z,t)$. Using this equation, we can replace soliton pulse voltage by their charge density profiles: $V_{j}=\frac{\rho_{j}}{a_{j}^{2}}$, where we have used a simplified representation of soliton voltage profiles that are solutions of the nonlinear Schrödinger differential equation. We can simplify the description further by writing $\rho_{j}$ as $\frac{Q_{j}}{\Delta_{j}}$ where $\Delta_{j}$ is an effective width of the pulse and $Q_{j}$ is the charge carried by the soliton. The speed of the effective soliton charge can now be written down from its profile. It is $v_{j}=\frac{a_{j}}{b_{j}^{3}}$ and is signal dependent. Given the equivalent charge profile and its speed, we can determine the magnetic field produced by it.

### 4.2. Solitons produce transient helical surface magnetic fields

The magnitude and direction of the field generated on the surface of a tube of radius r by these moving charge pulses can now be written down. The magnetic field has magnitude $B_{j}=\frac{Q_{j}v_{j}}{cr^{2}}$, in CGS units, where c is the speed of light in the medium, and its direction is tangential to the surface [29]. In terms of coordinates, the field is $B_{j}\cos(\frac{2\pi z_{j}}{r}),B_{j}\sin(\frac{2\pi z_{j}}{r}),0\leq z_{j}=v_{j}t\leq L$, where L is a loop length traversed by the pulse. For N moving soliton pulses, the magnetic field created, $\vec{B}$, is given by $\vec{B}=\sum_{j=1}^{N}\overset{\to }{B_{j}}$. This field has been measured [30] for a single action potential and used to accurately reconstruct it. Our next step is to work out the alignment of the surface spin-half particles due to this magnetic field. The dynamics of spin-half particles is governed by quantum theory. We will discuss the details of the structure shortly. For the moment, we state that a quantum theory calculation is necessary to study the problem and that the calculation shows that the magnetic alignment structure produced is a helical memory structure of spin-half particles. We now identify the nature of the spin-half particles involved and suggest where the structure is located. We next turn to address this issue.

### 4.3. Memory structures are aligned proton spins

Our network is required to have surface spin-half particles, either electrons or protons, in order to produce solitons, as both of these particles are present on the surface. We now give reasons why they must be protons, and we conjecture that the spin memory structure is located in the myelin sheaths of axons. The reason for the choice of protons is that although there are surface electrons in the cations Na^+^, K^+^ and Ca^2+^ and anions Cl^−^ in the electrolyte, all of these ions have a configuration where the 2*p* orbitals are occupied by two strongly spin-paired electrons. It is difficult to see how these electron spins could freely move to form structures. On the other hand, there are unpaired spins of the protons belonging to the hydrogen in water or in the membrane. There are many of them, but their moment is only 1/1800 of a Bohr magnetron. The small value of the Bohr magnetron makes their interaction very small. Thus, their polarization in the ionic current is even less than that of electrons, but they do have a long coherence time, since the nuclear spin–lattice relaxation time may be seconds. The transient helical spin structures might well be imprinted there.^7^

For this reason, we assume the memory structure comes from aligned proton spins. We then show that even though the Bohr magnetron for protons is small, the spin interactions are strong enough to form stable memory structures. We conjecture that the protons involved are located in myelin sheaths of axons.

There is some observational support for their conjectured location, which we now discuss.

### 4.4. Are myelin sheaths the location of memory structures?

The two fundamental supporting facts for considering proton spins as the building blocks of memory come from magnetic resonance imaging (MRI) measurements. These measurements show that proton spins can be aligned and that protons are present in myelin sheaths. It is also helpful that the relaxation times for MRI-aligned protons in myelin sheaths, the T1 relaxation time, is rather long $\approx 10^{3}$ ms. This long relaxation time will allow multiple soliton pulse magnetic fields, each of duration $10^{-3}$ s to contribute to creating the memory structure.

There is more support. Alzheimer, in 1911, had pointed out that the memory loss he observed was due to the degeneration of myelin sheaths. His observation was discarded for many years as the belief was that memories are stored in neurons, but now new research findings support Alzheimer’s original findings [33]. There is now considerable evidence linking memory and myelin sheaths. Here are some facts. It is found that preventing new white matter formation, myelin, hinders learning new motor skills, while structural changes [34] in white matter accompany motor training, namely the increase of fractional anisotropy revealed by diffusion tensor imaging methods of MRI after learning. These studies also show that the volume of white matter increases with learning, that the process of myelination is related to the learning of new skills, and that myelin sheaths support oscillations [35].

Furthermore, once myelin has formed, it is stable with little turnover of oligodendrocytes and limited remodelling of their lengths on existing myelin sheaths. Oligodendrocytes are the cells that myelinate the central nervous system. They are the end product of a cell lineage which has to undergo a complex and precisely timed programme of proliferation, migration, differentiation and myelination to finally produce the insulating sheath of axons. However, this stable structure may retain the capacity to remodel if myelin is disturbed [36]. Myelination begins prenatally and continues, in some areas of the brain, into middle age. The time course of myelination varies widely across brain regions. As a general rule, myelination occurs first in neural systems that underlie behaviours that are present early in life. For example, primary sensory and motor areas are myelinated before association areas, and the neural systems involved in postural control and the vestibular sense are fully myelinated before birth. On the other hand, areas of the prefrontal cortex do not become fully myelinated until middle age. By the age of 2, myelination is considered to be almost complete, except for the prefrontal cortex [37,38]. This might explain why our earliest memories begin after that age. It is found that myelin is composed of sheaths, with up to 100 layers for the peripheral nervous system, and they are spiral in the central nervous system axons [39]. The presence of multi-layers of myelin on an axon suggests how multiple memories may be stored on a single axon in a time-ordered way.

We next move to discuss the memory structure and prove that each structure has a memory-specific excitation frequency that may be used to recall the memory.

### 4.5. Mathematical representation of memory structures

We first focus on the memory structure and then determine its excitation frequency. Since spin-half dynamics is quantum, we have to use quantum methods to study these two features. A quantum calculation [40,41] (see electronic supplementary material) shows that the helical spin-magnet structure created by the transient pulse magnetic fields of moving solitons is given by


$$\begin{array}{r} S(z)=\left(\cos\left[\left(\frac{N_{c}\mu }{\bar{h}v}\right)N_{0}Bz\right]-i\sin\left[\left(\frac{N_{c}\mu }{\bar{h}v}\right)N_{0}Bz\right]\right)S_{L},\;0\leq z\leq L, \end{array}$$

where $N_{0}$ is the number of electrons in the pulse, $S_{L}$ is $S_{i}(0)$, the initial spin orientations values at z=0 of the $N_{c}$ proton spins located on a circle on the axon surface with centre z. These spins on a circle on the axon tube are aligned at the same time by the helical magnetic field produced by a soliton pulse. This explains the factor $N_{c}$ in the expression. The size of a memory will be estimated when we discuss the stability of the structure. There we will estimate the values of $N_{c},N_{0}$

The spin binding is greatly enhanced by the topological properties of spins that are under 2π rotation. Under such a rotation, the spin reverses its direction. This leads to a natural pairing of oppositely oriented spins at neighbouring site spins of the helix, as they are related by 2π rotations. Consequently, the memory structure produced has no macroscopic magnetic property. This is an unexpected result.

We next calculate the memory excitation frequency.

**Table:** 

variable	formula (CGS)	estimate
magnetic field $B_{j}$	$B_{0}=\frac{ev}{cr^{2}}$	$N_{1}0^{-9}gauss$
spin–field interaction H	$H=N_{s}N_{c}N_{0}\mu B$	$N_{s}N_{0}10^{-30}$ ergs
pulse $H\Delta t>\bar{h}$	$N_{s}N_{c}N_{0}\mu B10^{-3}>1$	aligned if $N_{s}N_{0}>10^{3}$
frequency $\omega_{j}$	$\omega_{j}=\frac{N_{c}N_{0}\mu B}{\bar{h}}$	$N_{0}$ Hz
spin–spin interaction $S_{ij}$	$S_{ij}=\frac{N_{c}\mu_{i}N_{c}\mu_{j}}{d^{3}}$	$10^{-12}>kT\approx 10^{-14}$
spin-topology W	$W=\sum_{i=1}^{k}[\alpha_{i}\beta_{i}]$	W=k, genus of engram

### 4.6. Memory excitation frequency

A quantum calculation given in the electronic supplementary material gives a simple formula for the memory excitation frequency. It is


$$\omega =\frac{H}{\bar{h}}=\frac{N_{c}\mu N_{0}B}{\bar{h}},$$

where $\bar{h}$ is Planck’s constant, μ is the magnetic moment of a proton spin, $N_{c}$ is the number of spins on a circle of axon radius at a given point of the axon and $N_{0}$ is the average charge carried by a single pulse. The value of this frequency will be estimated shortly. The range of this frequency depends on the charge carried by a soliton pulse and the profile of the magnetic field and hence is signal dependent. We also show that, due to the special topological properties of spin-half particles, there will always be a natural secondary memory structure present due to the pairing of two neighbouring spins of opposite orientations located at two neighbouring helical sites. The effective natural frequency of this predicted additional memory system is predicted to have double excitation frequency.

### 4.7. Memory stability

We now turn to discuss the stability of the memory structure and show that the memory structure of aligned proton spins created by solitons can be stable. The stability argument has three parts. First, it is shown that the weak magnetic field of moving solitons can align surface spins of protons. Then, we show that the transient magnetic field can create a stable structure. This discussion has several layers. The first layer shows spins can be aligned by the soliton’s magnetic field. The next layer has two parts: the first part shows that one strand of aligned spins is marginally stable, followed by a proof of the topological stability of the structure. Topology structure is essential; without it, the structure can be eliminated by smooth deformations and is possible. At this stage, the predicted topologically stable memory structure is in the aligned proton spins on myelin in the axons of pathways between a collection of neurons where topology stability requires the pathways must have loops. This structure looks like an engram. So, we call it an engram and estimate its size and the time it takes to form an engram. After these results are in place, we show that such a topological memory structure is stable under body temperature thermal fluctuations.

A helpful feature of the structure for stability is that it is held together by the interaction between oppositely aligned spin clusters.^8^

We show that not all signals produce stable memories. We listed three conditions required to create stable long-term memories. Let us go through them. The first condition is that the magnetic field $B_{s}$ must be able to align spins. The second condition is that the memory structure is topologically stable, and the third condition is that the structure is stable under body temperature fluctuations.

There are three numbers: $N_{0}$ is the average charge carried by a pulse, $N_{c}$ is the number of spins influenced by the magnetic field at one point z and $N_{s}$ is the number of soliton pulses that decide if the pulses can align spins, and one additional topological number, the spin topology number W is relevant for discussing topological stability of the structure. We first examine the alignment problem.

This requires the aligning energy pulse of duration Δ should be greater than $\bar{h}$. That is $\mu B_{s}\Delta t>\bar{h}$. We set $\Delta t\approx 10^{-3}$s, and $B_{s}=N_{s}N_{0}N_{c}B_{0}$, where the field $B_{0}=\frac{ev}{cr_{a}^{2}}\approx 10^{-9}$ gauss, where v is the speed of the signal, $r_{a}$ the axon radius, *v* is the velocity of light, $c\approx 10^{10}$cm s^−1^ is the speed of light and μ is the proton magnetic moment which is $\mu \approx 10^{-23}$ in CGS units. For our estimate, we have assumed that all pulses produce the same magnetic fields per unit charge, which we set to have the value $B_{0}$.

We can estimate $N_{c}$, the number of proton spins in a circle of radius equal to the axon radius $r_{a}$, thus: $N_{c}\approx \frac{2\pi r}{d}$, where d is the approximate separation between protons. We take $d\approx 10^{-8}$ cm and $2\pi r_{a}\approx 10^{-3}$cm. Then $N_{c}\approx 10^{5}$.

Putting in numbers, we get the alignment condition: $N_{s}N_{0}N_{c}\mu B\Delta t\approx N_{s}N_{0}10^{-30}>10^{-27}$ which gives the condition $N_{s}N_{0}>10^{3}$. Thus, a single pulse cannot align spins unless it carries $10^{3}$ charge units.

We next note that the memory excitation frequency has the theoretical value $\omega =\frac{N_{0}N_{c}B_{0}\mu }{\hbar }$. From this, it follows that for $N_{0}=10$, ω=10 Hz. If we identify these memory frequencies with sleep spindle oscillations observed, then the value of $N_{0}$ is in the range $10<N_{0}<20$. Thus, it is predicted that voltage pulses carry very small amounts of charge.

Using this result, we conclude that the minimum number of pulses $N_{s}$ required to align spins is in the range $N_{s}N_{0}>10^{3}$. We will call this a memory strand. It is not topologically stable.

We next discuss topological stability. Topological stability is essential for the following reasons. We would like our results to be relevant for a network that has not just the topological but the real, unknown, geometric connectivity of any brain connectome. This requirement is satisfied by the process of signal generation. Why is this condition important? It is important because if the memory structure is topologically stable, it will continue to exist unmodified by topological deformations so that results obtained from our simple topological description of the network remain valid for a network that has the geometric structure of a connectome. Such a smooth transformation exists from the topological classification theorem [23]. A simple condition ensures topological stability. A memory structure is topologically stable only if it has non-zero values for its spin topology number W.

### 4.8. Size of an engram

Recall that the spin topology number is defined as $W=4\sum_{i}[\alpha_{i}\beta_{i}]$, where i ranges over the genus of the signal-producing subunit and $\left(\alpha_{i},\beta_{i}\right)$ are theta function characteristics. The characteristic $\alpha_{i}$ means a traversal of a loop around the $a_{i}$ loop, and the characteristic $\beta_{i}$ means a traversal of a loop round the $b_{i}$ loop. It follows that a memory structure must have aligned spins that traverse both $\left(a_{i},b_{i}\right)$ loops for each value of the label i. This is a helical structure. However, a set of loops implies the presence of multiple neurons interlinked by axons to form multiple loops in an unknown way. Remember each $b_{i}$ loop needs to go through two junction points where each junction point is the location of a neuron. Only such an assembly of neurons linked together with loops will have a non-zero value for the spin topology number W and be topologically stable. Thus, topological stability requires a structure that describes an engram: a group of linked engram cells with spins arranged in a helical way. This is a theoretical prediction.

The value of W for a collection of engram cells has a topological meaning which is easy to identify. It can also be related to the number of neurons $N_{e}$ in the engram. If all $\left(\alpha_{i},\beta_{i}\right)$ are non-zero for i=1,2,…,k, then W=k and k is the effective genus of the surface of a specific assembly of engram cells while $N_{e}=W-1$. It gives the size of the engram.

A single link of aligned spins, with no loops, has W=0, also W=0 if either $\alpha_{i}$ or $\beta_{i}$ is zero. Thus, if the aligned spins do not form a helical structure, it will have W=0, and the structure is not topologically stable. We suggest that aligned spins with W=0 represent short-term memories.

### 4.9. Creation time for engrams

We next derive a formula for the time required to form a W≠0 memory structure. We showed that a memory link is thermally stable if the memory creating pulse numbers, $N_{s}$, is greater than $10^{3}$; this number assumes that each pulse carries just one unit of electric charge. With this result in place, we relate $N_{s}$ to time τ, the time required to produce $N_{s}$ pulses.

If we call the time required to produce $N_{s}$ pulses $\tau_{s}$ and the time required to produce one pulse t, then we can write $N_{s}=\frac{\tau_{s}}{t}$. We set $t\approx 10^{-3}$ s. Then, we have our formula for the creation time of a given memory link is $\tau_{s}=N_{s}10^{-3}$s. But topological stability requires multiple memory links that form loops so that the composite memory structure has spin topology number W that is non-zero. Putting these two results together, we have the time to form a memory engram $\tau_{e}$ is given by


$$\begin{array}{rl} \tau_{e}\; & =W\tau_{s},\\ \tau_{s}\; & =N_{s}10^{-3} \end{array}.$$

The number W is hard to determine, but it has a simple topological meaning. It is the effective genus k of a specific memory engram. The number $N_{e}$ of neurons in the engram is related to W by the formula $N_{e}=\left(W-1\right)$. Memory structures are interlinked, so that removing even a few special neurons can make W=0 and thus affect memory. The time required to form a complex memory formation will involve several independent brain units. For instance, for learning juggling, eye–hand coordination, balance and motor responses are relevant. Each one of these will involve neuron clusters $N_{i}$ to form the appropriate memory. When activated, the number of neurons firing will be $N=\Pi_{i=1}^{k}N_{i}$, where there are k classes of memories that form the engram.

### 4.10. Thermal stability of memory structure

We finally check that our third condition for stability is satisfied. This is the condition that ensures that the aligned spins in a given memory link can withstand thermal disruption when they are bound together by spin–spin interactions. This requires $E(s_{i},s_{j})\approx N_{c}\frac{(N_{c}\mu {)}^{2}}{d^{3}}>kT$. Putting in numbers $E(s_{i},s_{j})\approx \frac{10^{-36}}{10^{-24}}=10^{-12}>10^{-14}$. The condition is easily satisfied. The key input was that units of size $N_{c}$ are naturally aligned at the same time, and they thus interact as units.

It is an experimental challenge to either directly verify or rule out the predicted memory structure and the conjecture that it is located in the myelin sheaths of axons. We have provided some indirect evidence in support of the conjecture.

### 4.11. Magnetic resonance imaging magnetic fields do not destroy memories

We next show that MRI fields cannot destroy memories. For magnetic fields of a few tesla strength, the disruption is not strong enough to do this. The disruptive energy is $\mu B\approx 10^{-16}$ ergs, which is much smaller than the spin binding energy calculated earlier, namely, $\frac{(N_{c}\mu {)}^{2}}{d^{3}}\approx 10^{-12}$ ergs. The binding is between natural units of $N_{c}$ aligned spins. Thus, MRI events can slightly disrupt memories, not destroy them.

### 4.12. Table of numbers used to establish memory stability

The stability numbers used are summarized in the table. Values used are $N_{0}\approx 10,N_{c}\approx 10^{5},N_{s}>10^{3},c\approx 10^{10}$cm s^−1^.

### 4.13. Memory recall: similarity to quantum computer

Since memory structures have excitation frequencies, $\omega_{j}$, they could be recalled by a resonance excitation mechanism. The theoretical estimate for memory frequency values was found, remarkably, to overlap with those of observed EEG waveform frequencies. This suggests that memories could be recalled or consolidated by EEG waveforms [14].

The resonance mechanism of memory retrieval has similarities with the way a quantum computer works. In a quantum computer, the quantum evolution of a state contains in it all possibilities, and the process of identifying a specific possibility is in essence done by a resonance mechanism carried out by the overlap of wave functions, while here the memory space is a collection of different potential memory excitation frequencies encoded in topological stable patterns. An incoming signal with an associated set of EEG frequencies is then a probe which will excite and recall a set of memories by a resonance mechanism.

We next turn to discuss EEG waveforms.

## 5. Electroencephalography waveforms and their properties

### 5.1. Tiling waveforms

**Figure 8.**
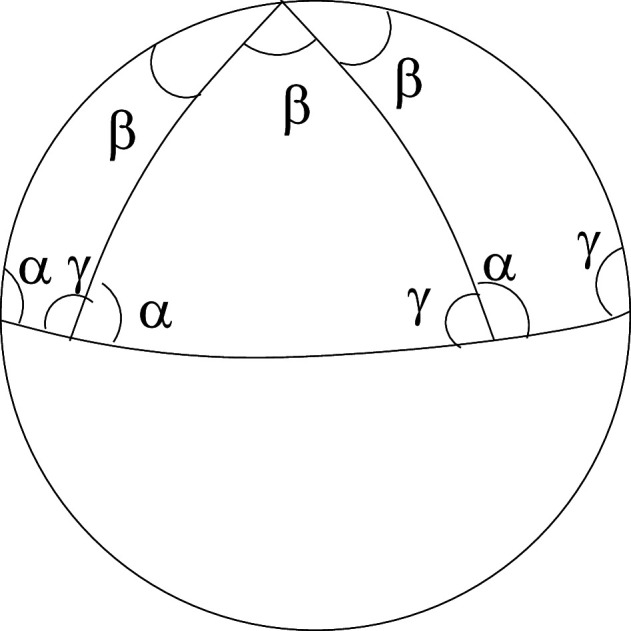
Schwarz tiling of a sphere with dihedral tiles of equal area. The spherical triangle with apex angle β and base angles $\alpha =\gamma =\frac{\pi }{2}$ and sides that are great circles tiles the sphere if the apex angle $\beta =\frac{\pi }{2n}$ where n is an integer. Possible tiling is related to Platonic solid surfaces projected onto the sphere. For example, an octahedron with eight triangular faces has n=4 and $\beta =\frac{\pi }{2}$, giving a tiling with eight triangular tiles, like the one illustrated.

Topological signal generation deforms signal-producing subunits of the Riemann surface to topological spheres. We will identify these topological spheres as the soma surfaces of neurons. It is expected that these surfaces will have surface voltage oscillations. We will prove the surprising result that the nature of the oscillations is determined by the spin topology number of solitons to be special waveforms that tile the surface of a sphere. This means that identical waveforms allowed can be placed together without overlap (figure 8) to tile the sphere surface. These results are proved using the fact that the oscillations are solutions of the wave equation on the surface of a sphere, not a topological sphere. Schwarz in 1873 had shown that tiling solutions on the surface of a sphere exist and that they must belong to five types of waveforms and are related to the existence of the five Platonic solids. Using our dynamic law, we will show that the topological sphere surface oscillations can be replaced by sphere surface oscillations, and thus the results of Schwarz and the link between soliton spin topology and tiling waveforms that we will prove can be used.

We now identify EEG surface waveforms as due to tiling waveform oscillations on an assembly of neuron surfaces. It is pleasing that their tiling nature is determined by the topological spin carried by solitons. We now show that the surface-tiling nature of the waveforms is enough for us to predict that EEG waveforms will have five frequency bands and determine their allowed frequencies and amplitude values. The prediction is compared to them with observations.^9^

This suggests that EEG waveforms are due to the surface oscillations of an assembly of neuron surfaces, which differs from a standard picture where dipole current oscillations from an assembly of individual neuron dendrites [13] are regarded as the source of EEG waveforms. The standard approach has nothing to say about EEG waveform frequency bands.

### 5.2. Comparison with experiment

Let us show how the tiling nature of waveforms determines their amplitudes and frequencies. If the waveforms tile the surface of a sphere by identical tiling waveform, then each waveforms will have a fixed area which is fixed by the number of tiling required to cover the sphere surface. A mathematical result tells us that there are only five such discrete symmetric tilings possible. They correspond to the Platonic solid surfaces. There is one extra infinite class of tiling that can be visualized as orange segment tiling. The number of segments can be arbitrary for this tiling. A simple intuitive argument can now be used to estimate the allowed frequency and amplitude values of the tiling wave forms. The area, $a_{2n}$, of a tiling solution of a sphere of radius R and area $A=4\pi R^{2}$ defines its wave form and is a measure of its amplitude. This result can be understood by considering a small deformation in the radial direction on the surface of a sphere of amplitude ρ. The total amplitude $a_{2n}$ of the deformation is then the sum of all these deformations over the tiling area. It is obtained by integrating the amplitude over one point of the sphere surface over the angles that define the tiling area. Thus, $a_{2n}=\rho \frac{A}{2nR^{2}}$, where angular dependence of ρ is ignored and R is the sphere radius. Now we use the intuitive formula $a_{2n}\nu_{2n}=c$ where $\nu_{2n}$ is the oscillating frequency of the tiling solution and c is the speed of the surface wave. Writing $a_{2n}=\frac{A}{2n}\lambda$, with $\lambda =\frac{\rho }{R^{2}}$, then gives $\nu_{2n}=2n\frac{c\lambda }{A}$, where $A=4\pi R^{2}$, R being the radius of the spherical neuron in its excited state. This shows the inverse relationship between the oscillation amplitude and its frequency. A list of our predicted values for frequencies and voltage amplitudes in table 1 is in agreement with observed EEG numbers [42], where the scale is fixed by choosing specific numbers for frequency and amplitude from those allowed for delta wave forms. The amplitude values are in a wide range compared with the frequency band ranges. Frequencies ν, in the table, are in hertz and the voltages are in microvolts. Besides the tiling of the sphere that comes from the five regular Platonic solids, mentioned in table 1, there is an additional tiling class for the sphere called the dihedral tiling with orange-segment-like tiles.

**Table 1.** The five Platonic solids and general dihedral tiling of a sphere with their predicted amplitudes and frequencies compared with those of EEG brainwaves [42]. Frequencies are scaled by the uniform excitation of the sphere.

solid	tile fraction	frequency	EEG range (Hz)	predicted amplitude (µV)	observed amplitude (µV)
dihedral	$\frac{1}{2n}$	≥2	all waves	$\frac{4\pi }{2n}$	All waves
sphere	$\frac{1}{1}$	1	delta (0.5−3)	200	100−200
tetrahedron	$\frac{1}{4}$	4	theta (4−7)	50	<30
cube	$\frac{1}{6}$	6	alpha (8−13)	33	30−50
octahedron	$\frac{1}{8}$	8	alpha (8−13)	25	30−50
dodecahedron	$\frac{1}{12}$	12	beta (14−30)	17	5−20
icosahedron	$\frac{1}{20}$	20	beta (14−30)	10	5−20
dihedral	$\frac{1}{40}$	40	gamma (31−50)	5	5−10

The area value of a given tile can be exactly determined by using a result of spherical geometry. If (α,β,γ) are the angles of a spherical triangle for a sphere of radius R, then its area A is given by the formula $\alpha +\beta +\gamma -\pi =\frac{A}{R^{2}}$[43]. If these angles are chosen to be $\left(\frac{\pi }{2},\frac{\pi }{2},\frac{\pi }{n}\right)$ then its area is $\frac{a_{2}n}{R^{2}}$, where two of these triangular sections are used to represent a wedge. These wedges tile the sphere surface into 2n pieces as stated earlier, with frequencies proportional to 2n. An analytic calculation [44] shows that the frequency is $\sqrt{(2n{)}^{2}-\frac{1}{4}}$. Thus, it supports the simple relationship proposed. The frequency numbers depend on the choice of scales. These are fixed by setting the frequency value of the delta waveform to be one and its amplitude to 200 μV. We note that dihedral tiling (orange segments) can produce all observed frequencies. An illustrative example is given that produces a specific gamma frequency. The analytic calculation [44] also gives an explicit expression for the tiling solutions that demonstrates their local nature, namely that a given oscillation is confined to one tiling segment.

The analytic way to determine the nature of sphere surface oscillations is to solve the sphere surface wave equation and impose suitable boundary conditions to produce tiling waveforms. This step requires replacing a topological sphere with a geometric sphere, which can be justified, as we will show, by using the dynamical principle of the network.

We now have a surprise. We find that the boundary conditions required to produce tiling waveforms are automatically fixed by the input soliton spin topology phases that enter a neuron to produce a signal. This is an unexpected result. It directly relates EEG waveform properties to topological properties of brain excitations. Let us prove this result.

### 5.3. Tiling waveforms are solutions of a wave equation

Recall a wave equation on any surface has the structure


$$\frac{1}{c^{2}}\frac{\partial^{2}\phi }{\partial t^{2}}-\nabla^{2}\phi =0,$$

where $\nabla^{2}$ is the Laplacian operator appropriate for the surface. The dynamical law requires all valid results of the surface must respect its underlying Riemann surface structure. The topological sphere is a Riemann surface that is conformal equivalent to a sphere [45]. Conformal symmetry is a basic property of any Riemann surface [24]. Thus, the dynamical law requirement that only results that respect the underlying Riemann space structure are valid means that only those results that are invariant under conformal transformations are valid. Since a topological sphere is conformal equivalent to a sphere, we solve the wave equation on the sphere and determine the allowed frequencies and waveforms. The oscillation frequencies found are invariant under conformal maps, but the Laplacian operator and waveform shapes are not. It follows that our results for frequencies are in agreement with the dynamical principle and are valid results, but waveform shapes obtained are valid within a conformal transformation.

We need one more mathematical detail. The Laplacian operator must have the symmetry of the sphere surface. This leads to the result that the radial part of the wave equation is the linear second-order hypergeometric differential equation [46], on the surface of a sphere, with three regular singular points [47]. We identify these three singular points as the location of three primary dendrites on a neuron surface. The median number of primary dendrites has been measured and found to be between three and four [48,49], and the range of primary dendrites observed varies between two and seven. We will consider the case of three dendrites. The analysis for the case when there are more singular points is possible [47].

### 5.4. Tiling waveforms are fixed by spin topology phase

The spin topology number phase is given by $e^{i\pi W}$, where $W=4\sum_{i=1}^{g}\alpha_{i}.\beta_{i}$ and $\left(\alpha_{i},\beta_{i},i=1,2,\ldots ,g\right)$ [19] are the discrete characteristics of the Riemann theta function, associated with the two classes of topological loop coordinates $\left(a_{i},b_{i}\right)$ of a Riemann surface (figure 3) where each characteristic $\left(\alpha ,\beta_{i}\right)$ can only take one of two values, namely $\left(0,\frac{1}{2}\right)$. It is a global property of the subunit and depends on all the loops of the subunit that generate a signal. The topological number has a simple geometrical meaning. It tells us the pathways involved in producing the soliton since the characteristic $\beta_{i}$ is associated with a loop $b_{i}$ while the characteristic $\alpha_{i}$ is associated with a loop $a_{i}$ of the Riemann surface (figure 2). The two characteristics thus define helical loops along the $\left(a_{i},b_{i}\right)$ loops of the Riemann surface. Notice that for W to be non-zero, the memory path should be helical.

Let us next explain why phases at singular points determine the nature of solutions. Suppose z=0 is a regular singular point and the solution of the hypergeometric equation looks like $Az^{\mu }$ near z≈0, where A is a constant and μ is not an integer. It is called the index of the singular point. If we replace z by $ze^{2i\pi }$, the solution changes from $Az^{\mu }$ to $Az^{\mu }e^{2i\pi \mu }$. If μ was an integer, the factor $e^{2i\pi \mu }=1$ but when it is not, we have a phase which describes the nature of the singular point. A mathematical result [47] tells us once μ is fixed, the nature of the corresponding solution of the hypergeometric differential equation [50] is determined.

We will now relate these singular point phases to the input soliton’s spin topology phases $W_{i},i=1,2,3$. Let us follow the procedure of Schwarz. The hypergeometric equation is a second-order differential equation and thus has two linearly independent solutions. Schwartz considered the ratio of the two linearly independent solutions that had the property of vanishing at each of its three regular singular points. The three singular points are the vertices of a triangle on the surface of a sphere. The vertices form a spherical triangle.

Then, solutions were constructed that vanish along the edges of the spherical triangle and showed that the angles of the triangles were given by the difference of the indices of the three singular points. Thus, if the angles related to the parameters of the hypergeometric equation were chosen appropriately, the solution waveform would be a localized oscillation within a region and would have the correct area to tile the sphere surface. Recall that for a spherical triangle, its angles fix its area. Let us write down these angles for the hypergeometric equation.

The hypergeometric differential equation is given by [50]


$$z(1-z)\frac{d^{2}y}{dz^{2}}+[c-(a+b+1)]z\frac{dy}{dz}-aby=0,$$

where (c,c−a−b,a−b) are real but not integers. The three singular points are (0,1,∞), where ∞ is the north pole of the sphere, and the points (1,0) are on the equator. For such a triangle, the angles of the solution triangle described were found by Schwartz to be π|1−c|,π|c−a−b|,π|a−b| corresponding to the points (0,1,∞).

Schwarz set $|1-c|=\frac{1}{p},|c-a-b|=\frac{1}{q},|a-b|=\frac{1}{r}$, where (p,q,r) were integers greater than or equal to 2, and showed that a spherical triangle with angles $\left(\frac{\pi }{p},\frac{\pi }{q}.\frac{\pi }{r}\right)$ tile the sphere provided $\frac{1}{p}+\frac{1}{q}+\frac{1}{r}>1$. There are only four possibilities: dihedral tilings where (p=2,q=2,r=2n) and n=2,3,…. The other tiles correspond to tetrahedral, octahedral and icosahedral tiles. As Yoshida [47] shows, these tiles can be related to the surfaces of Platonic solids, and the solutions are determined by these angles. We now show that input soliton spin topology phases entering at the three singular point locations, identified as dendrite locations, fix these angles.

Let us now write the angles of the solution triangles as phases $e^{i\pi t_{j}},j=0,1,\infty$ if $\left(t_{1}=\frac{1}{2},t_{2}=\frac{1}{2},t_{3}=\frac{1}{2n}\right)$, where n=1,2,… then these angle choices produce a dihedral tiling of the sphere. The corresponding phases are those associated with a solution of the hypergeometric equation near that singular point. We will call the phase $e^{i\pi t}$ as a twist angle.

These twist angles are fixed by the soliton spin topology phases: it is a dynamical mechanism. We assume that a unit twist W=1 associated with a soliton spin phase produces a twist of $e^{i\pi t}$ to a solution at a singular point of the hypergeometric differential equation. Then, a topological twist W produces a twist $(e^{i\pi t}{)}^{W}=e^{it\pi W}$. Next, we note that a set of characteristics is said to be even/odd depending on whether $e^{i\pi W}=\pm 1$ [24]. Solitons can have either odd or even characteristics, so the constraint of the topology phase they carry is simply $e^{i\pi W}=\pm 1$. For a given soliton, W can be either an even or odd integer.

Thus, we require $e^{it\pi W}=\pm 1$, that is $t=\frac{n}{W}$ where for a given soliton $\frac{n}{W}$ is either an even or an odd integer. For example, the set of values of t for the three regular singular points can be $\frac{1}{2},\frac{1}{2},\frac{1}{2n}$. This corresponds to the dihedral tiling example we used and corresponds to $W_{1}=2,W_{2}=2,W_{3}=2n$. The technical details are in Yoshida [47].

Fifteen explicit tiling solutions of the linear hypergeometric differential equation were discovered by Schwarz [51]. Later, a more comprehensive list of such solutions was found [52,53]. An example of a tiling waveform is shown in figure 4.

### 5.5. Special tiling waveforms: fractional Legendre functions

We now depart from discussing general tiling waveforms and restrict ourselves to the dihedral tiling. The advantage of doing this is twofold. First, explicit analytic expressions for these tiling solutions and their frequencies are known, and second, these solutions form a complete set. This means that any arbitrary function on a sphere can be expressed as a linear combination of these tiling solutions. They can thus be used to represent all other tiling solutions.

The analytic expressions for dihedral, cubic and tetrahedral tiling are known. They are fractional Legendre functions, $P_{\nu_{0}+2n}^{\mu }(z)$ [44]. These tiling solutions form a complete set so that they can be used to construct Green’s functions $G(z,t:z^{\prime },t^{\prime })$ [50]. With the help of Green’s functions, we can determine the output waveforms H(z,t) that will be produced in response to a given input $s(z^{\prime },t^{\prime })$. The mathematical details of the construction and the form of $G(z,t:z^{\prime },t^{\prime })$ are given in the electronic supplementary material. The completeness property also allows us to write any function f(z) on the sphere in terms of the fractional Legendre functions as


$$f(z)=\sum_{n=-\infty }^{i=+\infty }c_{n}P_{\nu_{0}+2n}^{\mu }(z),$$

where the points z represent points on arcs on the sphere. A slight generalization allows us to represent not just functions on the arc of the sphere but arbitrary functions on the sphere surface by considering the analogues of the usual spherical harmonics [50] for fractional Legendre functions. The ones related to dihedral tiling correspond to setting $\mu =-\frac{1}{2},\nu =-\frac{1}{2}$. They are


(5.1)Y−12+2n−12(θ,ϕ)=N(12,2n−12)e−iϕ28πsin⁡θ cos⁡(2n−12)θ,

where n=1,2,… and $N_{\left(-\frac{1}{2},2n-\frac{1}{2}\right)}$ is a normalization factor. They are evaluated in the next section. These functions are plotted for special values of N (figures 7 and 8) to show the two-dimensional tiling nature of these functions and that the solutions are localized. These functions also form a complete basis set of functions so that now any arbitrary function on the sphere surface can be represented using them.

### 5.6. Plots of electroencephalography waveforms in the dihedral approximation

**Figure 9.**
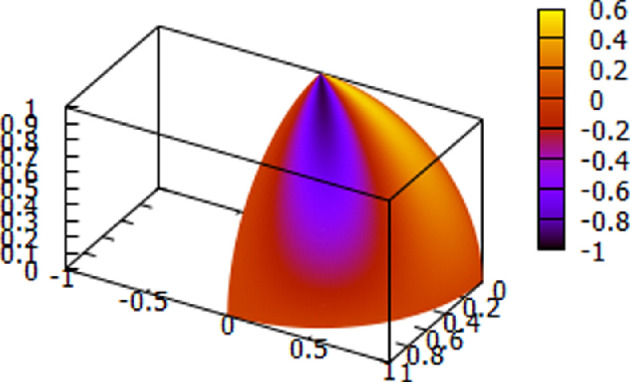
Theoretical tiling delta waveform using equation (5.2), *n* = 1. The figure shows how an assembly of neuron surface delta oscillations will appear in the brain, represented as a hemisphere of radius one. Note the oscillations are localized to a tiling sector of the hemisphere. The colour coding shows that the boundaries have zero amplitude.

**Figure 10.**
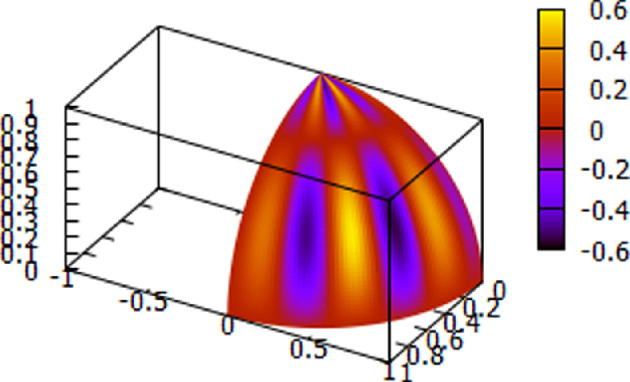
Theoretical tiling theta waveform using equation (5.2), *n* = 2. A second example. Note the tiling property.


Representative plots for the theoretically determined delta and theta waveforms are displayed using equation (5.2) in figures 9 and 10.

$$Y_{2n-\frac{1}{2}}^{-\frac{1}{2}}(\theta ,\phi )=N_{\left(-\frac{1}{2},2n-\frac{1}{2}\right)}\;e^{-i\frac{\phi }{2}}\sqrt{\frac{8}{\pi \sin\theta }}\;\cos(2n-\frac{1}{2})\theta ,$$

where n=1,2,…. The angles (θ,ϕ) are spherical polar coordinate angles with ranges (0≤θ≤π,−π≤ϕ≤+π). To construct tiling solutions that vanish on the sides of a spherical triangle, the following variable changes are required $\theta \to \theta^{\prime }=\frac{\theta }{2},\phi \to \phi^{\prime }=\frac{\phi }{2n}$ and we require that the solution be periodic in $\phi^{\prime }$. In terms of these variables, the solutions plotted are


(5.2)S2n−12−12(θ′,ϕ′)=1ncos⁡(nϕ′) 1sin⁡2θ′ cos⁡[(2n−12)2θ′]sin⁡θ′,

where the range of the angles is now $\left(0\leq \theta^{\prime }\leq \frac{\pi }{2},-\frac{\pi }{2n}\leq \phi \leq +\frac{\pi }{2n}\right)$. We check that this solution vanishes when $\theta^{\prime }=\frac{\pi }{2}$ and when $\phi^{\prime }=\pm \frac{\pi }{2n}$ which define the three sides of a spherical triangle. Thus, we have constructed a tiling solution.

### 5.7. Prediction of electroencephalography waves generated by input signals

The special Green’s function constructed in the electronic supplementary material can be used to determine the distribution of dihedral tiling EEG waveforms m(z,t) generated in response to any input signal s(t,z) by the formula


(5.3)m(z,t)=∫dt′∫dz′G(z,t,z′,t′)s(z′,t′),0≤t′≤t.

Consider two examples of input signals. Both are local signals, where one is a transient signal and the other an oscillatory signal. We have $s_{i}(t^{\prime },z^{\prime })=V_{0}\delta (z^{\prime }-z_{0}^{\prime })e^{-a_{i}t},i=1,2$ with $a_{1}=a_{0}>0,a_{2}=i\omega_{0}$. In the case of a transient input signal, delta waves and other oscillatory waveforms are produced, while in the case of an oscillatory input, if its frequency $\omega_{0}$ overlaps with any EEG waveform frequency $\omega_{n}$ then the predominant excitation output will be the EEG waveform of that frequency. This is an important remark. It tells us that transient high-energy input signals can produce slow oscillatory EEG waveforms.

The analytic solutions for the dihedral tiling waveforms, labelled by an index n, allow us to write down an analytic formula for their allowed frequencies [44]. We have,


$$\omega_{n}=\sqrt{2n^{2}-\frac{1}{4}},\;\;n=1,\ldots ;$$

however, the scale of these frequencies is not fixed by the equation. We have set the scale to be one. In electronic supplementary material, we use an energy argument which justifies this scale choice. The waveform shapes of these dihedral tiling solutions are given by the fractional spherical harmonics. They represent $\frac{V_{n}(\theta .\phi )}{V_{0}}$, the voltage of the waveform where $V_{0}$ is a scale to be chosen from the experiment. Thus,


(5.4)Vn(θ.ϕ)V0=Yn−12−12(θ,ϕ)(5.5)=N(−12,n−12) e−iϕ28πsin⁡θ cos⁡(n−12)θ,

where n=1,2,… and the real part of the harmonic is to be used. Thus, we have explicit expressions for surface waveforms and a formula for their allowed frequencies. The normalization factor $N_{\left(-\frac{1}{2},2n-\frac{1}{2}\right)}$ can be calculated by using Maier’s [44] results relating $P_{\nu }^{\mu }(\theta ),P_{\nu }^{-\mu }(\theta )$. Their norm $P=||P|{|}^{2}=\int_{{,}_{\pi }}^{+\pi }d\theta \;\sin\;\theta P_{\nu }^{\mu }(\theta )P_{\nu }^{-\mu }(\theta )$ can now be evaluated. We have


$$\begin{array}{rl} ||P|| & =\sqrt{\sqrt{\frac{2}{\pi }}\;\frac{\Gamma (\frac{3}{4})}{\Gamma (\frac{5}{4})}\;\frac{\left(4n+2\right)}{\left(2n+\frac{1}{2})(2n+\frac{3}{4}\right)}}. \end{array}$$

This result is required to calculate Green’s functions.

Two practical uses of EEG waveforms are given in the electronic supplementary material. They show how to represent an arbitrary surface function using the dihedral waveforms and how the location of a brain excitation can be unravelled from EEG waveforms.

However, these theoretical results simply use the mathematical idea of completeness and have limitations. Any input signal to the brain initiates brain processes, but since the brain is an open system, besides the input signal introduced, other internal or external signals will be entering the brain that will have consequences. These unknown deviations due to the open nature of the brain cannot be included in the theoretical formula. Consequently, the predicted mix of EEG waveforms for a given input will have deviations. In our view, these deviations provide insights regarding brain processes.

We next consider a sequence of EEG modulations observed during deep sleep and illustrate how the observed events can be understood as brain processes by the use of the properties of EEG derived. We then outline the way of the neural field model these events to highlight the differences between the two approaches.

## 6. Electroencephalography modulations observed in deep sleep

We consider a sequence of EEG modulations observed during deep sleep, the K-complex modulation, followed by sleep spindles, followed by sharp wave ripples [54]. We interpret these events as brain processes that consolidate and transfer memory. The account we give suggests that EEG waveforms play an important role in memory consolidation and transfer.

Sleep occurs in five stages: wake, N1, N2, N3 and rapid eye movement (REM) sleep. Stages N1 to N3 are non-rapid eye movement (NREM) sleep, with each stage leading to progressively deeper sleep. Approximately 75% of sleep is spent in the NREM stages, with the majority spent in the N2 stage. A typical night’s sleep consists of 4 to 5 sleep cycles, with the progression of sleep stages in the following order: N1, N2, N3, N2 and REM. A complete sleep cycle takes roughly 90 to 110 min. The first REM period is short, and as the night progresses, longer periods of REM and decreased time in deep sleep (NREM) occur [55]. The K-complex modulation appears in the N2 sleep phase and is followed by sleep spindles.

We interpret the observations as a sequence of brain events that are initiated by a major blocking excitation that leads to the K-complex modulation and follows through the expected consequences of this major modulation and shows this suggested chain of expected brain events explains sleep spindles and sharp wave ripples. We also make a general prediction. Signal blocking events, we believe, are essential for the functioning of the brain as they allow focused attention. Consequently, we expect such events to happen constantly at different energy scales and will show up in different EEG waveforms as sharp spikes [56]. Understanding the process of how they are triggered is a challenge.

### 6.1. Facts about K-complexes

In the N2 sleep cycle, delta waves are observed, and at intervals of 1.0−1.7 min, large sharp spikes, known as K-complexes, appear with amplitudes in excess of 100 mV. They modulate high-amplitude delta waves (1.6−4.0 Hz). The excitations have duration greater than 500 ms and are followed by spindle-shaped oscillations of delta waves, of duration a few seconds. The modulations are in the frequency range of 10–15 Hz and can be followed by sharp wave ripples [57–59]. Likely, the high voltage low-frequency waves are not delta waves but EEG waveforms generated by major blocking events that occur during deep sleep, since signal blocking excitations are known to result in EEG waveform production [60]. These waveforms are slow oscillating waves. We call them delta waveforms because all surface oscillations observed, in the network, come from neuron surface oscillations.

The K-complex sequence can be modelled in our approach in the following way: a major blocking event produces a modulation of a high-voltage delta wave which is then modulated by the K-complex event. The high voltage delta waveform then excites an underlying memory structure, and its excitation frequency modulation of the delta waveform produces the sleep spindle modulations, which then excite an underlying memory by a resonance mechanism that shows up on a delta wave as sharp wave ripples [61]. This sequence of events is an example of a feedback loop of information exchanges between signals mediated by EEG waveforms. It can be interpreted as a memory transfer from one brain location to another.

### 6.2. Transient localized signals generate K-complex

**Figure 11.**
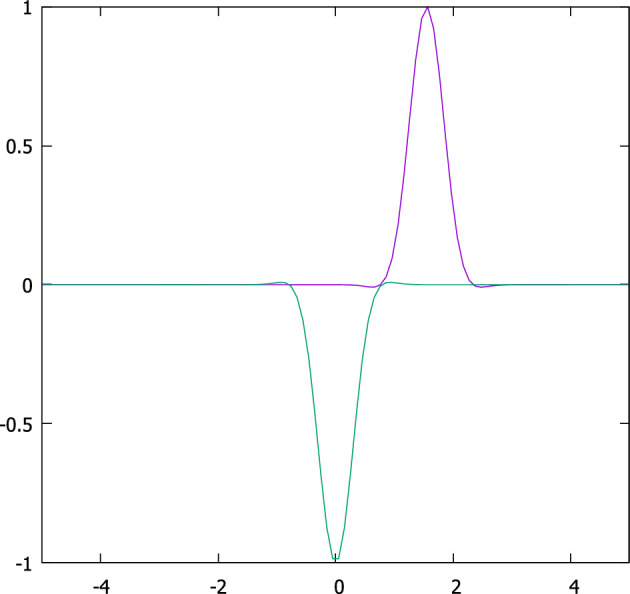
Theoretical K complex-like structure using equations (5.5) and (6.1)–(6.3). Scales are arbitrary.


Let us now give the mathematical details. Explicit analytic expressions for transient localized solutions of the nonlinear Schrödinger equation, that can cause signal blocking, exist [28]. To simplify our analysis, we approximate the known analytic solutions by the following simple expression:


$$\begin{array}{r} \psi (\theta ,\phi ,t)=\delta (\theta -\theta_{0})\delta (\phi -\phi_{0})e^{-(\omega_{n}t{)}^{2}}\Theta (t)-\Theta (t-\frac{1}{\alpha })\delta (\theta -\theta_{1})\delta (\phi -\phi_{1}), \end{array}$$

where $\left(\theta_{1}=\theta_{0}+\Delta ,\phi_{1}=\phi_{0}+\Delta \right)$, and Θ(t)=1,t>0 and zero otherwise, the Heaviside Theta function. For our qualitative discussion, we drop normalization factors and ignore the spatial oscillations of the waveforms. The theoretical surface waveform modulation due to a transient localized excitation is obtained by simply superposing this transient excitation voltage form with the delta waveform voltage. This is plotted in figure 11 using

(6.1)M(t)=M1+M2,(6.2)M1=−A1ne−(ωnt)2cos⁡ωnt, −T<t<+T,(6.3)M2=A1ne−(ωn(t−2T))2cos⁡ωnt, +T<t<+3T,

with $\omega_{n}=\sqrt{4n^{2}-\frac{1}{4}}$. For numerical work, an appropriate glueing function $M_{12}$ is required, and a value of the constant A has to be chosen. This is the K-complex. It is a modulation of a delta waveform by a transient localized blocking excitation.

### 6.3. Modelling sleep spindles

We next suggest that sleep spindles are induced modulations of delta waveforms due to the excitation of helical magnetic memory structures discussed earlier. The modulation occurs in a two-step process.

In the first step, the surface charges of the memory structure are acted on by the EEG waveform voltage. As the delta waveform voltage is high, this excites the memory to its natural frequency. This induced memory oscillation produces a potential ΔV that then modulates the EEG potential to produce a sleep spindle. Sleep spindles are thus interpreted as copies of memory structure frequencies.

We showed earlier, in equation (5.4), that the delta waveform potential, in the dihedral approximation, was


(6.4)V(θ,ϕ,t)=Ne−iϕ28πsin⁡θ cos⁡(2n−12)θ eiω0t,

where the time dependence of the oscillation is added and the voltage scale is included in the normalization constant N. The spherical polar coordinates have constraints $\frac{\pi }{2}<\theta <\pi$, and $\omega_{0}=\sqrt{(2n{)}^{2}-\frac{1}{4}},n=1,2,\ldots$. The gradient of −∇V is an electric field that acts on charges e on the surface, causing them to move. We replace $e^{-i\frac{\phi }{2}}$ by sin⁡(ϕ) and suitably adjust its range.

We next replace the (θ,ϕ) dependence of V(θ,ϕ,t) by a simpler expression, but we retain its theoretically determined time dependence. Thus, we set $V=V_{0}\sin\;\theta \;\sin\;\phi \;\sin\;t$ for the delta waveform, so that $\omega_{1}\approx 1$. Then, V(t)=V(x,y,t)=V(x,y,0)sin⁡t in Cartesian coordinates. Thus, we have electric fields induced by the delta waveform potential gradient in the *x* and *y* directions that acts on an electron of momentum $\vec{p}$. We have


$$\begin{array}{rl} \vec{E(t)}\; & =-\nabla V=\vec{E(0)}\cos t,\\ \frac{d\vec{p}}{dt}\; & =e\vec{E(t)},\\ \vec{p(t)}\; & =e\vec{E(0)}\sin t, \end{array}$$

where $\vec{E(0)}=-\nabla V$. This interaction causes a displacement of the electron and gives it energy. If this energy is high enough, it excites the memory structure, making it oscillate with its natural frequency $\omega_{0}=\frac{2\mu_{B}B}{\bar{h}}$ and an additional oscillation of frequency $2\omega_{0}$ that is due to the paired spin structures discussed earlier is also excited. To excite the memory structure, we require that $eE(0).ds>NE_{M}(0)$ where eE(0).ds is the electron energy corresponding to a displacement of the electron by ds, the memory structure energy is $E_{M}(0)=\bar{h}\omega_{0}$ and N is the number of electrons in the memory structure. Once this threshold is reached, the memory structure is excited, and each electron oscillates with energy $E_{M}(t)=\bar{h}\omega_{0}\sin\;\omega_{0}t$. We identify this oscillating energy term as the modulation voltage eΔV seen as a sleep spindle. We have


$$\begin{array}{rl} E_{e}(t)ds\; & =\frac{\vec{p(t)}.\vec{p(0)}}{2m}ds\\ \; & =\frac{\vec{p(0)}.\vec{p(0)}}{2m}ds\sin t,\\ E_{e}(0)ds\; & =\frac{\vec{p(0)}.\vec{p(0)}}{2m}ds\\ \; & \geq NE_{M}(t),\\ E_{M}(t)\; & =\bar{h}\omega_{0}\sin\omega_{0}t\sin t,\\ E_{e}(t)ds\; & =\mu_{B}.B\sin\omega_{0}t\sin t\\ \; & =e\Delta V. \end{array}$$

Thus, the modulation due to a single electron is given by $\Delta V=\frac{\mu .B}{e}\sin\;t\;\sin(\omega_{0}t),0\leq t\leq T$, while the observed modulation involves the cluster $N_{s}\approx 10^{3}$ that we found was necessary to form memories. We have used the result $\bar{h}\omega_{0}=\mu_{B}.B$. The modulation due to the original spin memory structure is thus


(6.5)ΔV=Nsμ.Besin⁡tsin⁡(ω0t), 0≤t≤T.

**Figure 12.**
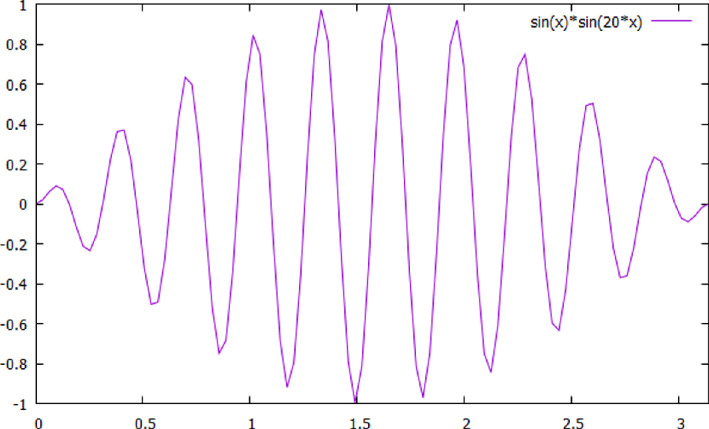
Theoretically determined sleep spindle structure with only the original memory structure considered equation (6.5). A second memory structure with double frequency and double amplitude is theoretically predicted. See text for details.

There is a corresponding $\Delta_{2}V$ modulation due to the induced electron spin-paired memory structure, given by $\Delta V_{2}=\frac{\mu_{B}.B}{e}\sin\;t\;\sin(2\omega_{0}t)ds,0\leq t\leq T$, where ω is the original memory structure frequency. The oscillation frequency of the memory has been written as sin⁡(ωt). The scale of the sleep spindle figure (figure 12) shown is arbitrary, and we have only plotted $\Delta V_{1}$.

Sleep spindles have observed frequencies in the range $\omega_{0}\approx 10\;-\;15$ Hz which is consistent with the theoretical estimate.

### 6.4. Interpretation of deep sleep events

The sequence of EEG modulations observed is interpreted as a mechanism of memory consolidation and memory transference from one region of the brain to another [62–64]. Major blocking events can create delta waveforms [60]. The network provides theoretical justification for such an interpretation. Our discussion of sharp wave ripples did not include details regarding them or provide an analysis of what inferences can be drawn from their observed features. We now provide these details. We then show how using our results regarding EEG generation and interpreting the sharp wave ripple as a modulation caused by memory transference signals leads to the estimate of the number of neurons firing that accompany it.

### 6.5. Comments on sharp wave ripples

Let us state some facts about sharp wave ripples. They modulate delta waveforms by frequencies in the ranges 80–1500 Hz, and also in the ranges 20–40 Hz and by one of frequency ≈5 Hz. Sharp wave ripples are a major brain event that can involve between 50000 and 100000 neurons firing [65]. They follow sleep spindle events.

From the network perspective, a resonance-induced signal, like all brain excitations, is topologically produced and thus can be described by a pinch deformation. This means EEG waveforms will be co-produced. The nature of these EEG waveforms, as we showed, is determined by the unknown spin topology numbers w of the generated signals. We recall how this works using a dihedral tiling to illustrate the process. Recall that three input spin topology numbers $\left(w_{i},i=1,2,3\right)$ entering the three primary dendrites of a neuron fix the nature of oscillations on the neuron surface. For dihedral tiling, these three input numbers are (2,2,2n) and they produce an EEG waveform of oscillating frequency ω=2n. Thus, by observing EEG oscillations, the spin topology number w of a signal can be determined.

The value of w also, as was pointed out, reflects the size of a memory circuit, its effective genus k which in turn is related to the number of neurons $N_{k}$ that define the circuit. We have w=k and $N_{k}=\left(w-1\right)$. Thus, to create frequencies in the range 80–150 Hz, we need (80<w<150). Similarly, to produce the frequency range 20–40 Hz, we need spin topology numbers in the range 20<w<40 and for ≈5 a w≈5.

We now assume that the observed EEG waveform modulations in a sharp wave ripplet are related to memory engram neurons firing. This will involve engram neurons for the different sensory memories. From the observed sharp wave ripple EEG waveforms, we can extract the following memory engram numbers: $\left(N_{v}\approx 10^{2},N_{a}\approx 10^{2},N_{t}\approx 10,N_{s}\approx 5\right)$, where $N_{v}$ is the vision engram size, $N_{a}$ is the audio engram size, $N_{t}$ is the touch engram size and $N_{s}$ is the smell engram size. Multiply these numbers, and we get an estimate of the expected number $N_{E}$ of firing neurons. We get $N_{E}\approx 50\;000$.

This sketch suggests how observational data can be interpreted using the network’s vocabulary. Thus, for sharp wave ripples, we used the EEG frequencies observed to predict the expected number of firing neurons. We have related the high-frequency EEG components with vision and audio senses and the lower frequency EEG waveforms with touch and smell.

Our prediction is that any structure supporting memory should exhibit the frequency ranges similar to those found for sharp wave ripples as they reflect memory recall and transfer processes. The memory structure is in the pathways, but the excitation frequencies are triggered by the firing of neurons that are linked together by these pathways to form an engram. The associated EEG waveforms co-produced during this process contain in them the firing neuron details that create them.

We now briefly comment on the difference between the network approach to model brain events and that of neural field theory by examining how neural field methods model the K-complex sequence. In the network approach, complex processes of the brain are related to a sequence of elementary processes that involve the interplay of brain signals, EEG waveforms and memory structure using biology and physics inputs. The inclusion of feedback loops of interactions between memory and signals is a key process. In our discussion of K-complex events, this approach was used. This was possible because the network suggests how EEG waveforms are related to other brain signals, suggests how and where memories are stored, and suggests a way EEG waveforms can interact with memories.

In neural field theory, all brain processes involve a competition between inhibitory and excitatory neurons that is process-dependent. This powerful insight, coupled with biology-driven interactions, allows neural field theory to model a variety of brain processes. The focus in neural field theory when modelling the K-complex sequence of events is to selectively understand specific excitations observed, such as slow wave oscillations and K-complex [59], sleep spindles [66,67] and memory transfer [68]. There is also difficulty in formulating the problem [59] as K-complex events are modulations on an elevated voltage background. EEG waveforms are introduced as induced excitations due to dipole current loops on dendrites. Such a model does not produce the observed frequency amplitudes of EEG waveforms and does not suggest how interactions between signals and memory occur. Finally, in modelling memory transfer, the neural field theory approach does not relate memory transfer to observable events such as sharp wave ripples but to long-term age-related degeneration of memory.

## 7. Discussion

In this article, a different topological way of thinking about brain signals, how they are created and how memories are formed is suggested. Signals were generated by pinch deformations of axon tubes. This idea has biological support [69]. Pinch deformations are the input signals of the network and can be created by electric, mechanical or chemical means. The mathematical parameters that describe pinch deformations come from the Riemann structure of the surface network. Thus, the process of signal generation is autonomous. We also explained the way memory structures of aligned proton spins could form and be recalled by their memory-specific excitation frequencies.

### 7.1. Fundamental brain processes

The central idea is that global topological features of the brain may provide insights that are otherwise not available. To implement this global approach, we used mathematical ideas of topology, algebraic geometry and physics. However, even though the mathematical ideas used are unfamiliar, we emphasize that the new approach offers an intuitive way of thinking about brain processes. The approach suggests that all brain processes involve a small number of elementary sub-processes such as blocking signals, resonance interactions between EEG waveforms and memory, modulation of waves and induced feedback loops between signals and memory. In this list, understanding blocking signals and how they may be generated by a person by chemical means is a key feature. Blocking signals isolate a system so that it can focus on specific events. It is an essential feature of all cognitive functions of the brain. Each subprocess has an intuitive meaning. We showed how interactions between memory and signals could be described and intuitively understood.

We next summarize some of the key results established.

### 7.2. Electroencephalography creation

The suggested method of generating EEG waveforms as special surface voltage oscillations on spheres, created by pinch deformations, immediately links them to these pinch deformation-created excitations and shows that they tile the surface of a sphere. From this, we can predict their frequencies and amplitudes. Surprisingly, the frequency bands of EEG waveforms overlap with the memory excitation frequencies predicted theoretically. This is surprising because the origin of the two frequencies is very different: one is a classical system describing surface oscillations, while the other is a result of a quantum system acted on by a magnetic field. This overlap of frequencies, however, plays an essential role in the functioning of the brain, as it allows EEG waveforms, co-produced with an incoming signal, to excite memories by the mechanism of resonance excitation and thus identify its nature.

Two further practical results are derived and placed in the electronic supplementary material because of their technical nature. One predicts the mix of EEG waveforms expected in response to an arbitrary input voltage signal, and the other reveals the EEG structure of an observed brain excitation event. The first result is expected to have deviations for two reasons: first, new brain events occur during the period of observation, and second, if the input signal initiates additional brain excitations that are not represented in the mathematical scheme. Both these deviations thus throw light on the way the brain functions and make the procedure a useful probe. The second reveals the extent of the event, that is, the number of neurons involved in its creation.

A major result of the approach was to predict a memory structure that had structural features similar to engrams but with the additional prediction that the memories were in the pathways between neurons.

### 7.3. Engram location and properties

Memory structures naturally emerge in the surface network. The first step is that pinch deformations generate signals that carry information, are charged, and by the laws of physics produce magnetic fields that in turn act on surface spin-half particles to create topological memory structures with memory-specific excitation frequency. We conjectured that the memories are stored in helical aligned proton spins with non-zero spin topology number W, located in myelin sheaths of axons.

A non-zero spin number means that the structure must include loops of aligned spins, but loops in the network have neurons at their junction regions. Thus, the theoretical topological stable memory structure predicted is one of a group of neurons linked together by pathways with non-trivial topology, where the memories are in the pathways. This predicted structure has the features of the observed memory engram.

We derived a formula for the creation time τ for an engram and its size in terms of the spin topology number W. An engram can encode multiple memories. Each memory engram can encode a specific subset of memories and will have its own non-zero $W_{e}$ value. The creation time τ for such an engram we showed is given by the formula


$$\tau =W_{e}N_{s}N_{0}t,$$

where $N_{s}$ is the number of pulses required to create a thermally stable memory strand, $N_{0}$ is the average charge carried by a pulse and t is the time required to generate one pulse. We found the condition for aligning spins was $N_{s}N_{0}>10^{3}$. By identifying sleep spindle frequencies with memory excitation frequency, we found $N_{0}\approx 10$. Thus, we choose $N_{c}\approx 10^{3}$ as the number of pulses that can stably align spins. Finally, we set $t\approx 10^{-3}$ s as a measure of the time taken to create one pulse. Our memory creation time then becomes $\tau \approx W_{e}$ s. The spin topology number $W_{e}$ can be related to the number of neurons $N_{e}$ in the memory unit, its size, by the formula $N_{e}=\left(W_{e}-1\right)$. But this time, the estimate does not include the additional time required to myelinate axons, if necessary for storing the new memories.

Aligned spins with W=0 that do not form loops are not topologically stable memories. We interpret them as short-term memories. Their lifetime is expected to be related to the relaxation time T1 of MRI for white matter.

We conjectured that the memory structure is located in myelin sheaths of axons. We offered some evidence that this may be the case. We now make an additional prediction in support of the conjecture. If myelin sheaths do store memories, then they should be involved in producing sharp wave ripples. Recall that we interpreted sharp wave ripples as a signature of a memory transfer event. Results established for the networks predict that the nature of observed EEG frequencies is determined by the three spin topology numbers $w_{i},i=1,3$ of signals entering a neuron through its three primary dendrites. The value of $w_{i}$ depends on the number of neurons $n_{e}$ involved in the process and is given by $\left(n_{e}=w-1\right)$. For dihedral tiling, the three spin topology numbers are (2,2,2n) and they produce an EEG frequency ω=2n. These frequencies for the sharp wave ripple were 80–150, 20–40 and 5 Hz. These EEG frequency values are expected to be present in all memory structures of the brain and hence should be present in memory recall and transfer processes involving engram neurons.

### 7.4. Brain excitations can be chaotic

In our discussions, all excitations considered were non-chaotic [10]. However, chaotic brain excitations can be produced in the system in two ways. The first is by the coupling of two oscillatory modes of different frequencies. For example, two large brain excitations, such as a K complex, close together can couple EEG waveforms co-produced to produce chaotic excitations. It is known that two coupled oscillators, a double pendulum, can produce chaotic behaviour.

The second way is if the surface network has defects. By defects, we mean points or regions of surface discontinuity. If, for example, the axon is narrowed by scars or there are local periodic structure discontinuities due to connectivity changes, then additional excitations can result. Such effects can be modelled by perturbations of the nonlinear Schrödinger equation by impulses of short duration at these special locations or by introducing local periodic structure differences as potentials. It is known that chaotic excitations can be produced in the nonlinear Schrödinger equation by perturbations of this type [70].

## 8. Conclusions

In this article, a surface network with special properties was studied. The network suggests testable solutions to a number of long-standing issues in neuroscience research. The first issue is that although it is established that memories are stored in engrams, the questions of how memories are stored and in what form they are stored are unanswered. The surface model addresses this issue by suggesting that memories are aligned proton spin structures located in the myelin sheaths of axons. The axon pathways are between the assembly of neurons, and the memory structure they support is stable only if the pathways include loops.

The second issue is how memories are recalled. In the surface model, the memory structure is a quantum spin system with each memory having a specific excitation frequency. Thus, a given memory may be recalled by a resonance mechanism which excites its memory from an assembly of memories. This process was discussed, and it was pointed out that it was similar to the way a quantum computer works.

The next issue is conceptual, and it suggests how a system creates its unknown communication code. The surface network suggests a way. The parameters of input pinch deformation signals that define the information content of a signal were shown to a self-generated communication code.

The next issue is to explain the origin and predict the properties of EEG waveforms, namely why they have five frequency bands. This issue was addressed by relating EEG waveforms to special oscillations on the surface of an assembly of neurons, induced whenever other brain signals are generated. The suggested mechanism correctly predicts the observed frequency bands of EEG waveforms and remarkably relates them to the existence of the five Platonic solids of antiquity.

We now summarize some of the predictions of the approach. The most significant prediction is that memories are aligned proton spins, located in myelin sheaths of axons that belong to a cluster of neurons with non-zero spin topology numbers.

(1) A strong prediction made was that soliton signals carry with them information regarding the way they are created. This information is encoded in the network’s own code, which is given by the unknown pinch deformation parameter values that generated the signal.
(2) The example of thalamic neuron spikes discussed provides evidence that multi-soliton solutions of the nonlinear Schrödinger equation can reproduce observed brain action potentials, and the fits confirm the theoretical prediction that each spike is different.
(3) We expect that action potentials from the different sensory organs that look the same are different in detail and expect that their nature (visual or auditory) can be determined from these details because they are created by different types of pinch deformations. Using machine learning methods, it should be possible to test this prediction.
(4) The oscillation frequency bands and amplitude values of EEG waveforms as special sphere surface oscillations were predicted and were found to be in reasonable agreement with observations. Recent observations support the idea of neuron soma oscillations when action potentials are emitted. It is observed that the neuron soma is deformed when the neuron emits an action potential. The sequence of deformations observed was successfully modelled by treating the neuron soma as a sphere [26]. The observed surface also has ridges that may represent localized tiling waveforms.
(5) In a recent paper, Pang *et al.* [71] were trying to find the best global marker of brain activity. They chose solutions of the equation $\nabla^{2}\psi_{\lambda }=-\lambda^{2}\psi_{\lambda }$, where the Laplacian operator $\nabla^{2}$ is that of a sphere surface and found that these solutions provided the best marker for brain activity. This result confirms the expectation of the network. In the network, EEG waveforms are special solutions of $\nabla^{2}\psi_{\lambda }=-\lambda \psi_{\lambda }$ and they are predicted to be the best markers of brain activity as they are co-produced whenever any brain signal is created by topological means.
(6) Since EEG oscillations are suggested to be due to oscillating neuron soma surfaces, they should be the same for all creatures with neurons. This prediction is confirmed for a very wide range of vertebrates, including fish, amphibians, reptiles, birds and mammals, large and small by Bullock [72] and for human beings with different mental disorders by Fingelkurts [73].
(7) It is predicted that EEG waveforms are modulated by other brain events, and these modulations themselves can lead to new excitations. We used these ideas to interpret the sequence of EEG modulations that follow a K-complex event, as a process of memory consolidation and transfer [4]. The special feature of this analysis is that it includes interactions between memories and incoming signals via EEG waveforms in a feedback loop.
(8) The presence of spin structure is an essential part of the network dynamics. Pre-existing spin structures of the brain are expected to be present from birth. Hence, memory formation, which requires modifications of spin structure, does not start from a blank slate, but builds on a pre-existing structure [74].
(9) The memory structure is predicted to have a natural, theoretically estimated frequency, and it is also predicted that a second associated memory structure with double this excitation frequency should be present due to memory structure spin pairing. Such a double frequency has been experimentally observed [75]. This frequency doubling is important as it means low-frequency gamma waves can also excite the memory [14].

The method of EEG waveform generation proposed here differs from those currently proposed [13,76–80] in an essential way. Here, EEG waveform creation is directly related to a coherent process of surface oscillations of a large number of neurons, each producing a mix of the distinct classes of surface oscillations discussed, whenever excitations are generated in response to pinch deformations described in this article. But in all existing theories, action potential generation and EEG waveforms are not directly related.

### 8.1. Future directions

The focus of the paper was on a method of generating all brain-like signals. As we proceeded, it became clear that the approach naturally suggested a way for storing memory, and it suggested that the brain may operate using a small number of elementary processes, each one of which is shown in the network to have a mathematical representation. Underlying all of them is the one key process: the selective blocking of signal pathways. In neural field theory, this fundamental feature is built in as the balance between inhibitory and excitatory neurons for each brain process. In the network, we need to find a global mechanism that does this. In our discussions, we introduced transient localized excitations as a way of doing this. Such signals exist and can be generated by pinch deformations, and they are thus globally generated. However, what triggers them needs to be understood. We suspect the answer lies in the trigger signals induced by hormone/chemical/gas releases in the brain. These blocking events allow focused attention and are thus vital for all cognitive functions of the brain.

In view of these remarks, the simulations of brain activity in the network should involve a sequence of linked elementary brain processes, each of which has a mathematical representation. The path of activity is expected to be driven by the environment and the intentions of a person and will involve thresholds set by feedback loops of interactions between memory and signals and between signals and hormone/chemical releases. These feedback loops are space-time dependent. A sketch of how such an approach would work was given in the description of the K-complex sequence of events. But a general operational scheme is lacking.

The immediate challenge is to observationally confirm or rule out our suggested structure for memory and its conjectured location. The current work is the start of a new global way of thinking about the brain that might be useful.

## Data Availability

Supplementary material is available online [81].

## Footnotes

^1^
We summarize some of the results of our paper [19] that we need to discuss EEG waveforms and the way they interact with memory.

^2^
The brain connectivity architecture evolves. Thus, a connectome changes. Our results hold for a changing connectome as well.

^3^
When neurons produce action potentials, it is observed that their surface is deformed to a more spherical shape, and they have surface deformations [26].

^4^
The magnetic field generated by an action potential has been measured and has been used to accurately reconstruct the original voltage pulse signal [30]. Thus encodes all the information carried by the signal.

^5^
Helical spin-half structures suggested here are possible and have been observed in condensed matter physics [31].

^6^
The technical details of the deformed Fay identity are omitted. They are discussed in a separate paper [19].

^7^
The argument is due to Mike Coey, personal communication.

^8^
The pairing of neighbouring spins makes the pair a boson so that they can cluster together unaffected by the Pauli exclusion principle's repulsive effects. Thus, it increases the stability of the structure.

^9^
There is experimental evidence that the soma of neurons becomes more spherical when they emit action potentials and have observed surface ridges that have been measured [26].
